# Convergent Evolution of Escape from Hepaciviral Antagonism in Primates

**DOI:** 10.1371/journal.pbio.1001282

**Published:** 2012-03-13

**Authors:** Maulik R. Patel, Yueh-Ming Loo, Stacy M. Horner, Michael Gale, Harmit S. Malik

**Affiliations:** 1 Division of Basic Sciences, Fred Hutchinson Cancer Research Center, Seattle, Washington, United States of America; 2 Department of Immunology, University of Washington School of Medicine, Seattle, Washington, United States of America; 3 Howard Hughes Medical Institute, Fred Hutchinson Cancer Research Center, Seattle, Washington, United States of America; University of Bath, United Kingdom

## Abstract

The ability to mount an interferon response on sensing viral infection is a critical component of mammalian innate immunity. Several viruses directly antagonize viral sensing pathways to block activation of the host immune response. Here, we show that recurrent viral antagonism has shaped the evolution of the host protein MAVS—a crucial component of the viral-sensing pathway in primates. From sequencing and phylogenetic analyses of MAVS from 21 simian primates, we found that MAVS has evolved under strong positive selection. We focused on how this positive selection has shaped MAVS' susceptibility to Hepatitis C virus (HCV). We functionally tested MAVS proteins from diverse primate species for their ability to resist antagonism by HCV, which uses its protease NS3/4A to cleave human MAVS. We found that MAVS from multiple primates are resistant to inhibition by the HCV protease. This resistance maps to single changes within the protease cleavage site in MAVS, which protect MAVS from getting cleaved by the HCV protease. Remarkably, most of these changes have been independently acquired at a single residue 506 that evolved under positive selection. We show that “escape” mutations lower affinity of the NS3 protease for MAVS and allow it to better restrict HCV replication. We further show that NS3 proteases from all other primate hepaciviruses, including the highly divergent GBV-A and GBV-C viruses, are functionally similar to HCV. We conclude that convergent evolution at residue 506 in multiple primates has resulted in escape from antagonism by hepaciviruses. Our study provides a model whereby insights into the ancient history of viral infections in primates can be gained using extant host and virus genes. Our analyses also provide a means by which primates might clear infections by extant hepaciviruses like HCV.

## Abstract 2

Escape from antagonism by hepatitis C and related viruses has repeatedly evolved in antiviral factor MAVS via convergent evolution, revealing an ancient history of previous viral encounters in primates.

## Author Summary

Hepatitis C virus (HCV) causes chronic liver disease and is estimated to infect 170 million people worldwide. HCV is able to establish a persistent infection in part by inhibiting the innate immune response. It does so by using its protease, NS3, to cleave the host's antiviral factor MAVS, which normally activates the interferon response. Using an assay that measures MAVS activity, we found that multiple primate species contain a version of MAVS that is resistant to HCV antagonism. Surprisingly, most of these primates have independently converged on changes in the same amino acid residue of MAVS to escape cleavage by the HCV protease. We found that the HCV protease has lower binding affinity for these resistant MAVS variants, which consequently are more effective at restricting HCV infection. Using a combination of phylogenetic and functional analyses of proteases from other HCV-related viruses, we infer that ancestral primates were likely exposed to and adapted to HCV-like viruses. One consequence of this adaptation is that changes that have given rise to extant MAVS variants may now provide protection from modern-day viruses.

## Introduction

Among the myriad of antiviral mechanisms employed by mammalian cells, the ability to sense viral RNA has emerged as a critical component of innate immunity. Viral RNA is detected in the cytoplasm by sensor proteins RIG-I and MDA-5 [1]–[3]. Both these sensors act through a common downstream effector Mitochondrial antiviral signaling (MAVS) (also known as IPS-1, VISA, and Cardif), which in turn mounts an interferon response (schematic of MAVS pathway in Figure S1) [4]–[7]. Given the importance of the RNA virus-sensing pathway in the antiviral response, it is not surprising that several highly diverse classes of viruses have evolved ways to inhibit multiple steps of the viral-sensing pathway.

In particular, several viruses encode antagonists of MAVS function [5],[8]–[13]. Hepatitis C virus (HCV) encodes a protease NS3, which in concert with its cofactor NS4A cleaves human MAVS [5],[8]. HCV is a single-stranded positive sense RNA virus that belongs to the *Flaviviridae* class of viruses that causes chronic liver disease and is estimated to infect 170 million people globally (about 3% of the world's population) [14],[15]. HCV is known to naturally infect only humans, although chimpanzees can also be experimentally infected with it. GB viruses GBV-A, GBV-B, and GBV-C are other HCV-related viruses belonging to the *Flaviviridae* class that infect primates, although it is not clear whether they are pathogenic to their hosts. We refer to all GB viruses as hepaciviruses in this article, although some GB viruses have also previously been referred to as Pegiviruses (the International Committee on Taxonomy of Viruses has not yet formally assigned GB viruses to any genus) [15]. Recently, HCV-like viruses have also been found in non-primate mammalian species, specifically in bats and dogs [16],[17].

We investigated the functional consequences of MAVS evolution, putatively driven by antagonism from ancient viruses (paleoviruses). Viral antagonism can impose persistent selective pressure on host antiviral factors like MAVS, resulting in positive selection (i.e., accumulation of an excess of nonsynonymous changes relative to synonymous changes over evolutionary time). Positive selection has been seen previously in many antiviral factors characterized in primate genomes [18]–[24]. Positive selection largely reflects adaptations to past viral infections, with adaptive changes providing beneficial consequences for the host to overcome viral antagonism. However, the resulting adaptive changes can also influence resistance or susceptibility to present-day viruses. For example, adaptive changes at key residues in the antiviral gene Protein Kinase R (PKR), likely driven by ancient viruses, are important determinants of PKR's ability to resist antagonism by present-day poxviruses [18],[25]. Thus, antiviral genes evolving under positive selection are good candidates to be genetic determinants of resistance or susceptibility to present-day viruses.

Here, we sought to determine whether MAVS has adaptively evolved during primate history and whether this positive selection has consequences for its resistance against HCV antagonism. We found that MAVS has evolved under strong positive selection in primates. We further found that one consequence of this selection is that MAVS from multiple primate species has independently become resistant to HCV antagonism. Remarkably, most of these primate MAVS variants are resistant due to changes at the same residue, and we dissect the functional consequences of this change using a combination of biochemistry and virology. Finally, based on functional analysis of other extant HCV-like viruses, we infer that these adaptive “escape” changes in MAVS were likely driven by ancient hepaciviruses, potentially providing paleoviral insights into hepaciviral infections over the course of primate evolution.

## Results

**Figure 1:**
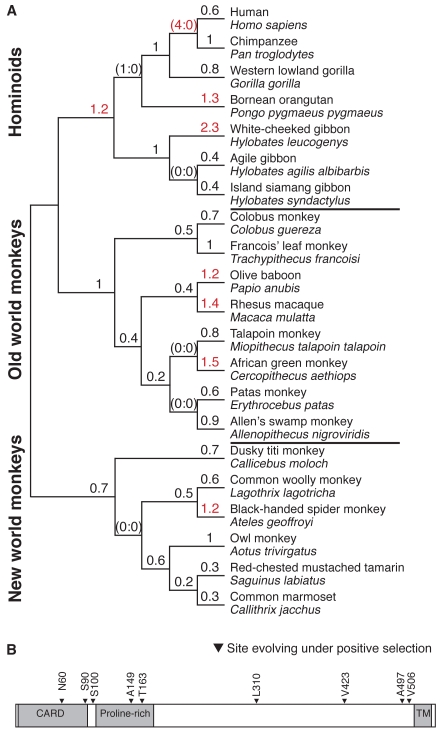
MAVS has evolved under positive selection in primates. (A) dN/dS ratios for the MAVS gene, calculated using free-ratio model in PAML, are shown on a phylogeny [26] along with common and scientific names of the primate species represented. Branches with dN/dS ratios greater than 1 are highlighted in red. For branches that did not experience any synonymous (S) changes, number of nonsynonymous (N) and S changes are shown in parentheses. (B) Residues identified as being under recurrent positive selection are indicated on a schematic of the MAVS protein, including the CARD, Proline-rich, and transmembrane (TM) domains. CodeML program of PAML software was used to identify sites under positive selection by comparing M7 (beta) versus M8 (beta & ω) models, assuming codon frequencies according to the F61 model. Web-based implementation of HyPhy package was used to perform REL analysis. We present only those sites identified with a posterior probability higher than 0.9 in both Bayes empirical Bayes (BEB) and random effects likelihood (REL) methods.

We sequenced and analyzed MAVS cDNA from a total of 21 simian primate species representing nearly 40 million years of simian primate evolution (Figure 1A) [26]. The phylogeny constructed with MAVS sequences was in agreement with the recently published well-supported phylogeny of primates [26]. We found that many residues in MAVS were highly conserved throughout the course of 35 million years of primate evolution, reflecting the constraint to maintain MAVS function via purifying selection. However, using maximum-likelihood analyses [27],[28], we found that MAVS has evolved under strong positive selection in primates. Specifically, models that allow the rate of nonsynonymous (dN) changes to be greater than that for synonymous (dS) changes are statistically a much better fit to the data than models that disallow positive selection (*p*<0.001, Table 1). Although there appears to be considerable variation in dN/dS ratios across numerous branches of the MAVS phylogeny (Figure 1A), according to branch-site random effects likelihood (REL) analysis, no single branch has statistically significant evidence of positive selection (*p*>0.05) [29]. Indeed, models that allow dN/dS to be variable along branches do not have a statistically higher likelihood than models that account for one common dN/dS ratio across the entire phylogeny (*p*>0.05, Table 1) [27],[28], suggesting that positive selection is not confined to a few primate lineages but is rather pervasive throughout the primate phylogeny. In addition to inferences about adaptive evolution across the entire MAVS protein, we can infer positive selection at the single amino acid resolution based on recurrent nonsynonymous mutations at a single position across multiple lineages (dN/dS>1 at a single residue). According to maximum likelihood analysis using PAML [27],[28], 10 residues have high posterior probabilities of having dN/dS greater than 1 (>0.9). Nine of these 10 residues are also supported by an independent random effects likelihood (REL) analysis (Figure 1B, Table S1) [30]. These findings of positive selection are consistent with the hypothesis that MAVS has encountered and adapted to viral antagonists throughout primate history.

**Table 1:** Pervasive adaptive evolution of MAVS in primates.

Selection Models	Codon Frequency Model	Degrees of Freedom	−2 X lnL λ	*p* Value	% Sites with dN/dS>1 (Average dN/dS)
M7 (beta) versus M8 (beta & ω)	F61	2	42.5	*p*<0.0001	7.7 (4.4)
	F3X4	2	39.9	*p*<0.0001	7.8 (4.5)
M8a (beta & ωs) versus M8 (beta & ω)	F61	1	41.3	*p*<0.0001	7.7 (4.4)
	F3X4	1	39.3	*p*<0.0001	7.8 (4.5)
M0 (fixed ω) versus M1 (variable ω)	F61	38	26.2	0.93	N/A
	F3X4	38	30.7	0.80	N/A

M7 is a null model in which dN/dS (ω)>1 is not allowed. M7 assumes 0<ω<1 is beta-distributed among sites, while M8a assumes 0< ω≤1. These null models are compared to selection model M8 with an extra category that allows sites with ω>1 in likelihood ratio tests. These comparisons support evolution of MAVS under positive selection in primates. Models M0 and M1 are free-ratio models that are compared to test for branch-specific positive selection. M0 model allows all branches to have the same common ω, while M1 allows each branch to have its own ω. This comparison does not support episodic selection of MAVS in primates. All analyses were done using codeml program in PAML software.

**Figure 2:**
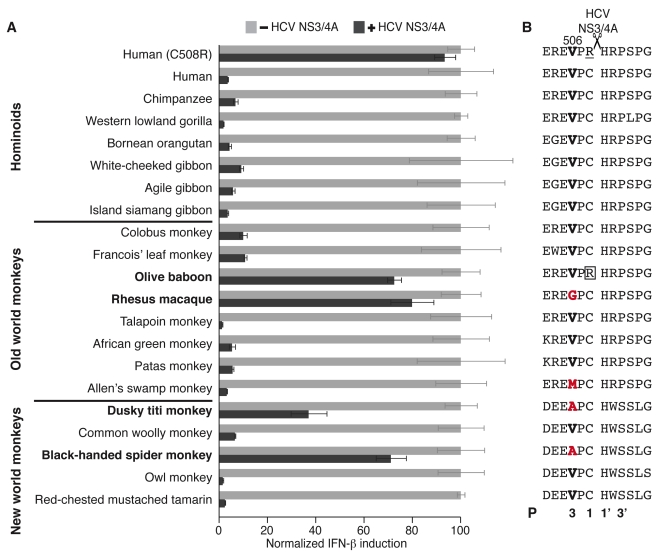
MAVS from multiple primate species is resistant to antagonism by HCV protease NS3/4A. (A) Induction of IFN-β promoter, as measured by luciferase firefly activity, upon expression of MAVS cDNA from corresponding species coexpressed with (+) or without (−) HCV NS3/4A. Primates with MAVS capable of significant IFN-β induction even in the presence of HCV protease are highlighted in bold. Human (C508R) refers to substitution of Cysteine (C) at position 508 with Arginine (R) in human MAVS. Firefly luciferase activity is normalized as being 100% for MAVS from each species in absence of HCV NS3/4A. All experiments are done in triplicates, and error bars indicate standard deviation. (B) Amino acid sequence of MAVS (residues 503–514) from corresponding primates in (A). The scissor icon indicates HCV NS3/4A cleavage site. The P positions for the HCV protease are indicated at the bottom of the alignment. Arginine (R) at residue 508 in olive baboon, believed to prevent MAVS cleavage, is boxed and the C508R change in human MAVS is underlined. Position 506, highlighted in bold, correlates with the ability of primate MAVS to induce IFN-β even in the presence of HCV NS3/4A protease as seen in (A). Changes away from the ancestral valine (V) at position 506 are indicated in red.

Given the rapid divergence of primate MAVS via positive selection, we investigated whether MAVS alleles from different primate species harbored functional variation in terms of their susceptibility to antagonism by extant viruses. For instance, if primates have encountered and adapted to escape antagonists similar to HCV protease, then MAVS from some of these primate species might be resistant to inhibition by HCV NS3/4A. We tested this possibility using a facile assay to measure HCV NS3/4A inhibition of MAVS activity. Over-expression of human MAVS in cultured cells is sufficient to activate the interferon response, which can be assayed by the IFN-β promoter-driven firefly luciferase reporter. We cloned MAVS from 20 different primate species into expression vectors with a strong CMV promoter and transiently transfected them into human-derived 293T cells. We found that MAVS from all primate species was capable of inducing IFN-β-driven luciferase expression, despite relying on the signaling machinery of human cells (Figure 2A, Figure S2). Consistent with previous observations [5],[8], we observed that co-expression of HCV NS3/4A protease robustly inhibited human MAVS signaling to the IFN-β promoter (Figure 2A). This inhibition is specific to the HCV protease-mediated proteolysis at residue 508, since introducing a C508R mutation in human MAVS blocks this inhibition, as shown previously (Figure 2A) [8]. We found that HCV NS3/4A also inhibited signaling driven by MAVS from most primates. However, MAVS proteins from four species—olive baboon, rhesus macaque, spider monkey, and dusky titi monkey—were capable of significant IFN-β induction even in the presence of the HCV protease (Figure 2A). Except for olive baboon and rhesus macaque, all the “resistant” species are phylogenetically well separated from each other by “susceptible” species (Figure 1A). Thus, resistance to inhibition by HCV protease appears to have been independently acquired at least three different times.

We next investigated the molecular basis of MAVS resistance to HCV NS3/4A inhibition. We found that MAVS from olive baboon has the same cysteine to arginine change at position 508 as in our positive control MAVS with C508R mutation (Figure 2B), disrupting the protease cleavage site and thus providing an explanation for its escape from HCV protease inhibition. However, the cysteine at 508 is conserved in other “resistant” MAVS' (Figure 2B), suggesting that genetic changes at different residue(s) must be responsible for their resistance to HCV protease. By examining the MAVS protein sequences from rhesus macaque, spider monkey, and dusky titi monkey, we found that changes at only a single residue, 506, can parsimoniously explain MAVS escape from HCV protease antagonism in these variants (Figure 2B). While most primate MAVS proteins have an ancestral valine residue at position 506, the three HCV protease “resistant” MAVS' have all independently altered this residue to either glycine or alanine. We therefore considered the remarkable possibility that MAVS has converged on the same escape strategy multiple times in primate evolution. Consistent with escape from viral antagonism being responsible for this convergent evolution, residue 506 is one of nine MAVS residues with high statistical support of having evolved under positive selection (Figure 1B, Table 1).

**Figure 3:**
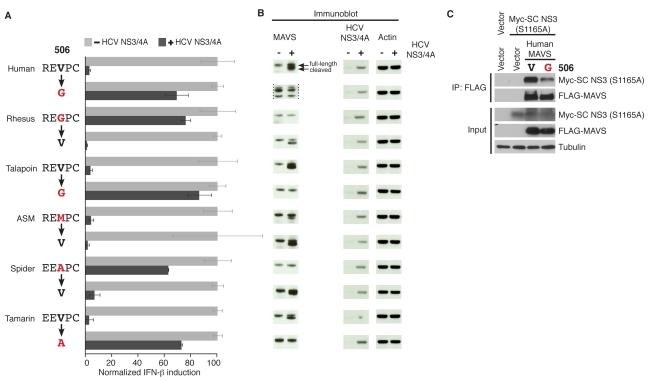
Changes at residue 506 in MAVS protect it from cleavage by HCV NS3/4A protease. (A) Normalized induction of IFN-β promoter by MAVS variants with the indicated changes, as measured by luciferase firefly activity, is presented in the presence or absence of HCV NS3/4A protease. Evolutionary changes in amino acid at position 506 are highlighted in red. Firefly activity is normalized to be 100% in absence of HCV NS3/4A. Experiments are done in triplicates, and error bars indicate standard deviation. (B) Immunoblot analysis showing MAVS, NS3/4A, and actin protein from cells expressing the MAVS variant with or without NS3/4A according to corresponding labeling in (A). The human V506G MAVS variant was expressed at low levels and is therefore shown at higher exposure (marked by dotted lines). Lowest size bands are non-specific in these samples. (C) Co-immunoprecipitation analysis of MAVS with the single-chain (SC) HCV NS3/4A protease containing the protease active site mutation S1165A. Extracts from HEK 293 cells expressing Flag-tagged human MAVS with either valine or glycine (in red) at position 506 and Myc-SC-NS3 (S1165A) were immunoprecipitated using Flag-conjugated beads followed by immunoblot analysis.

To assess whether changes at position 506 are causal for resistance to HCV NS3/4A protease, we swapped the evolved glycine and alanine residues in rhesus macaque and spider monkey MAVS, respectively, back to the ancestral valine (G506V and A506V, Figure 3A). We found that both of these swapped MAVS variants were capable of IFN-β induction and were now susceptible to HCV NS3/4A inhibition (Figure 3A). Conversely, changing the ancestral valine in human and talapoin MAVS to glycine (V506G) and to alanine (V506A) in tamarin monkey MAVS confers significant resistance to NS3/4A protease antagonism (Figure 3A). Taken together, these data demonstrate that changes at residue 506 in MAVS are necessary and sufficient to explain most of the variation in resistance and susceptibility to the HCV NS3/4A protease among primate MAVS proteins. However, not all changes appear to be equally protective. For instance, Allen Swamp Monkey has a methionine instead of the ancestral valine at position 506 but is still susceptible to inhibition in the luciferase assay (Figure 2B, Figure 3A) (but see below).

Residue 506 occupies the P3 position of the NS3/4A protease cleavage site, only two residues away from where the protease cleaves MAVS (Figure 2). Previous studies have demonstrated that this position is an important determinant of HCV polyprotein cleavage by NS3/4A protease, with valine being the preferred residue at P3 [31],[32]. However, functional effects of variation at the P3 position for MAVS cleavage were previously unknown. We investigated whether “resistant” changes at residue 506 protect MAVS from cleavage by the viral protease (Figure 3B). We found that “resistant” rhesus macaque and spider monkey MAVS' are not cleaved by the NS3/4A protease (Figure 3B). However, single amino acid substitutions back to ancestral valine render both rhesus macaque (G506V) and spider monkey (A506V) MAVS' susceptible to cleavage. Conversely, V506G changes in human and talapoin MAVS and a V506A change in tamarin monkey MAVS render these previously susceptible variants to now become resistant to NS3/4A protease cleavage (Figure 3B). Intriguingly, we also found that Allen's swamp monkey MAVS, which possesses a methionine at position 506, becomes more susceptible to cleavage when the methionine is changed back to the ancestral valine (M506V) (Figure 2B, Figure 3B), suggesting that methionine at 506 does offer protection even though this was less evident from the IFN-β induction assay (Figure 2A, Figure 3A). Thus, cleavage susceptibility to HCV NS3/4A protease explains the variation we saw in the IFN-β induction assay (Figure 2A, Figure 3A).

To investigate the mechanism underlying cleavage resistance, we tested the importance of residue 506 (position P3) for stable complex formation between NS3/4A and MAVS. To measure stable interaction between NS3/4A and MAVS, we used the catalytically inactive protease to prevent cleavage and release of MAVS from a MAVS/NS3/4A complex. As the protease catalytic activity is needed to cleave the protease polypeptide into NS3 and its cofactor 4A, which is required for proper NS3 function, we used a single chain protease construct of NS3 that contains an active site mutation to prevent MAVS cleavage, but contains the amino acids of NS4A required for maximal protease activity fused to the amino terminus of the NS3 protease (sc-NS3 (S1165A)) [33]. We observed reduced binding of the HCV protease to human MAVS when the “susceptible” valine at residue 506 is mutated to “resistant” glycine (Figure 3C). These data suggest that reduced binding between MAVS and the protease contributes to protection from cleavage of MAVS variants with “resistant” changes at position 506.

**Figure 4:**
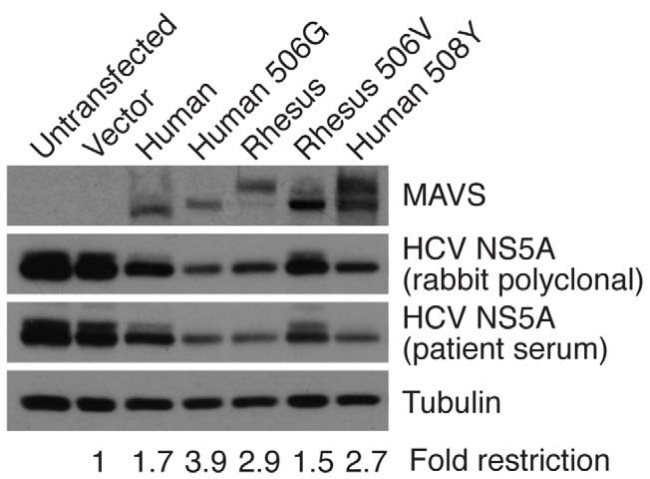
Protease-resistant MAVS better restricts HCV replication. Immunoblot showing HCV NS5A protein from lysates of Huh7-K2040 cells transiently transfected with control plasmid (vector alone), human, or rhesus MAVS variants with indicated mutations. The amount of HCV restriction achieved by indicated MAVS variant is quantified below the Western blot.

Next, we tested whether resistance to cleavage by the HCV protease allows MAVS to restrict HCV replication with greater efficiency during an infection. Cell lines that harbor subgenomic HCV replicons provide a good model system to study HCV replication [34]. Importantly, replication of this HCV genome is susceptible to IFN-induced host responses [35]. We therefore used a Huh7 cell line that harbors a HCV type 1b–derived replicon to test whether cleavage-resistant variants of MAVS are better able to restrict HCV replication [34]. Transient transfection of wildtype human MAVS confers a nearly 2-fold higher protection against HCV replication in cells (Figure 4). Moreover, transfection with cleavage-resistant human MAVS (V506G) or rhesus MAVS confers an additional 2-fold higher restriction of HCV replication, which is equivalent to that achieved by complete loss of MAVS cleavage due to mutation at the P1 position (C508Y) (Figure 4). The actual level of resistance is likely underestimated in these experiments since not all cells exposed to HCV replication were expressing MAVS, which was transiently transfected. We conclude from these experiments that resistant variants of MAVS are better able to restrict HCV replication, with changes at residue 506 (P3 position) allowing MAVS to achieve the same level of HCV restriction as the non-cleavable MAVS with mutation at the P1 position.

**Figure 5:**
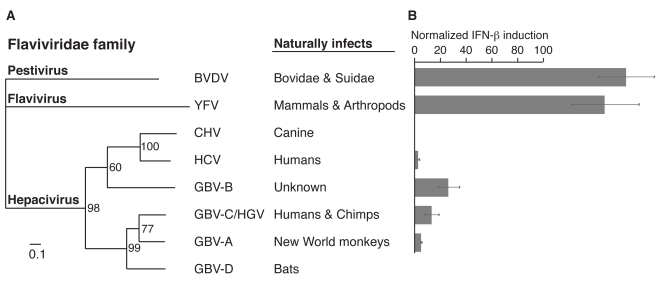
NS3 proteases from distantly related GB viruses inhibit human MAVS. (A) Phylogram made using NS3 sequence from the indicated viruses. Bootstrap values are indicated for each branch. Scale represents 0.1 amino acid substitutions per site. Bovine viral diarrheal virus (BVDV) and Yellow fever virus (YFV) are representative pesti- and flaviviruses, respectively. We refer to GBV-C and GBV-A viruses as hepaciviruses, although they have not been formally assigned to a particular genus within the Flaviviridae family. GBV-C is also known as Hepatitis G virus (HGV). CHV stands for canine hepacivirus [17]. Corresponding hosts that can be naturally infected by each virus are indicated on the right. (B) IFN-β induction, as determined by luciferase firefly activity upon expression of human MAVS in the presence of NS3 protease from the corresponding virus in (A). IFN-β induction is normalized as being 100% in absence of any protease. Experiments were performed in triplicate with error bars indicating standard deviation.

Statistical support of positive selection based on convergent evolution in four independent lineages suggests that changes at residue 506 represent adaptations driven by viral antagonists. Functional analysis demonstrates that changes at this residue now protect MAVS against cleavage by protease from an extant virus HCV, allowing MAVS to better inhibit HCV replication. Taken together, these data are consistent with the hypothesis that changes at residue 506 represent adaptations to antagonism by proteases from HCV-like viruses. To support this hypothesis, we wished to test proteases from viruses that infect non-human primates that might antagonize MAVS at the same position as the HCV protease. The GBV-B virus is closely related to HCV and has been tentatively assigned to the Hepacivirus genus. And although its natural host is unknown, it has been shown to infect tamarins and marmosets [36],[37]. More distantly related to HCV are the GBV-A and GBV-C (also known as hepatitis G) viruses, which are known to naturally infect primates. GBV-A infects New World monkeys while GBV-C is found in humans and chimps. These two viruses have tentatively been assigned to a new Pegivirus genus, as a sister genus to the Hepacivirus genus within the Flaviviridae family (although this designation has not yet been formally accepted by the International Committee on Taxonomy of Viruses) [37]–[42]. We refer to the Hepacivirus/Pegivirus genera as hepaciviruses here, for convenience and because of their shared properties we observed (Figure 5). All GB viruses encode the NS3 protease. We therefore tested the ability of NS3 proteases from these viruses to antagonize human MAVS. Consistent with our hypothesis, we found that the NS3 protease from both GBV-A and GBV-C strongly inhibited signaling by human MAVS (Figure 5B). Consistent with previous reports, NS3 protease from GBV-B also inhibited human MAVS, while proteases from the more distantly related pestivirus bovine viral diarrhea virus (BVDV) and flavivirus yellow fever virus (YFV) did not antagonize MAVS (Figure 5) [43]. Thus, the property of antagonizing MAVS is shared and exclusive to the group of viruses encompassing the HCV and GB viruses.

**Figure 6:**
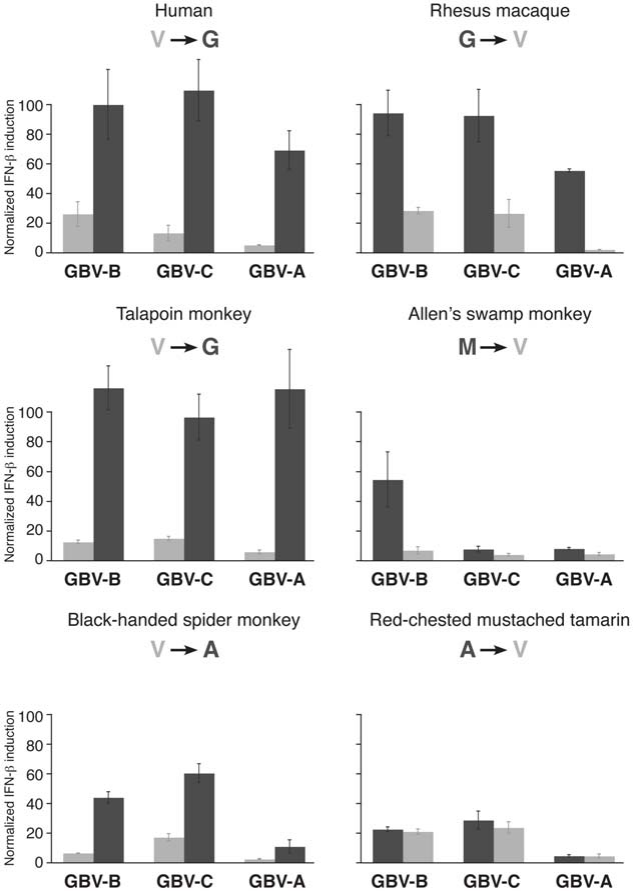
Protective effect of changes at residue 506 on antagonism by GB virus proteases. IFN-β induction resulting from expression of MAVS from indicated species, as determined by luciferase firefly activity, is presented for either the ancestral “susceptible” valine (light grey) or derived “resistant” variants (dark grey) at position 506 as shown. Luciferase firefly activity is normalized as being 100% in absence of the protease for each MAVS variant. Susceptibility to each of the GB-virus protease is shown. *x*- and *y*-axes are the same for all graphs. All experiments were performed in triplicate with error bars indicating standard deviation.

We next tested the effect of positively selected changes at residue 506 on the ability of the GB viruses to inhibit MAVS. Remarkably, changes at residue 506 away from ancestral valine in MAVS allow it to signal in the presence of NS3 proteases from all GB viruses (Figure 6, Figure S3). However, there are some species- and virus-specific differences. For example, methionine at residue 506 in Allen's swamp monkey MAVS appears to provide significant protection against protease from GBV-B virus compared to other GB viruses and HCV (Figure 2A, Figure 6). Also, alanine at residue 506 in red-mustached tamarin monkey did not provide a much higher protection against antagonism by GB viruses (compared to HCV, Figure 2A) than the ancestral valine. These idiosyncratic differences likely reflect slightly different properties of the HCV and GB virus proteases. However, our overall data suggest that the ability of NS3 proteases to antagonize MAVS, and to be impeded by the same adaptive changes in MAVS, is a phylogenetically discrete characteristic exclusive to HCV and the three GBV viruses, and likely inherited from a common ancestor. Importantly, these data demonstrate that non-human primates are susceptible to infection by viruses that antagonize MAVS in a manner similar to HCV.

**Figure 7:**
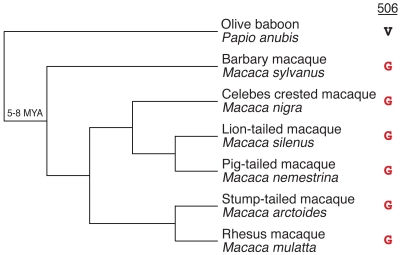
Mapping the adaptive change at site 506 in the Macaque lineage. Accepted Macaque phylogeny. Both species names and common names are shown. Amino acid at position 506 in MAVS is shown on the right. The escape variant glycine (G) is shown in red. Note that the outgroup Olive baboon has valine (V) at position 506. This indicates that the common ancestor of macaques underwent the adaptive change 5–8 million years ago. MYA, million years ago.

To infer the minimum age of viruses that drove the evolution at residue 506, we wanted to date these adaptive changes. We took advantage of the phylogenetically well-characterized macaque lineage, which includes rhesus macaque with the “resistant” change at position 506 [44]. We sequenced the C-terminus of MAVS from six different *Macaque* species and found that all of them, including the most ancestral species, the barbary macaque *M. sylvanus*, possess the “resistant” glycine instead of the ancestral valine at position 506 (Figure 7). Furthermore, we found no polymorphisms at this position within 20 rhesus macaque samples, suggesting that the change to glycine is likely a fixed change within the species. Since the macaque lineage separated 5–8 million years ago from that of baboons [44], which retain the ancestral valine at position 506, our data suggest that the progenitor macaque lineage encountered and adapted to a virus that antagonized MAVS at least 5–8 million years ago.

## Discussion

In this study, we have shown that positive selection in MAVS of multiple primate species has independently resulted in its escape from antagonism by hepaciviral proteases. Including baboons that have acquired a change at residue 508, we found that 5 out of the 20 primates that we characterized possess MAVS resistant to hepaciviral antagonism. Remarkably, MAVS resistance in four of these species is a product of independently acquired changes at the same single residue 506, which allow escape from hepaciviral NS3 protease recognition and cleavage.

### MAVS as a Genetic Determinant of Hepaciviral Clearance

HCV cleaves MAVS in order to block the interferon response. The importance of this strategy employed by HCV is highlighted by the fact that interferon treatment is often clinically used to successfully cure HCV infection. Furthermore, a polymorphism near a type III interferon gene is associated with spontaneous and treatment-induced clearance of HCV [45],[46]. Thus, given the importance of the interferon response, we hypothesize that primates that possess MAVS resistant to cleavage by NS3 protease should be better at clearing hepaciviral infections than species with susceptible MAVS. Consistent with this idea, we found that resistant variants of MAVS are better able to restrict HCV replication in cell culture. Furthermore, although sampling of hepaciviruses from primates has been sparse, thus far hepaciviruses have only been found in primates with “susceptible” MAVS, such as human, chimp, common marmoset, red-chested mustached tamarin, and owl monkey [38]. In contrast, hepaciviruses were assayed for but not found in spider monkeys, which have “resistant” MAVS [38]. Our study provides functional evidence that implicates host genetics in determining the outcome of diverse hepaciviral infections even between closely related primate species.

### Ancient Hepaciviruses as Drivers of MAVS Evolution

The remarkable case of convergent evolution at residue 506 is indicative of adaptive evolution, likely driven by ancient viruses (paleoviruses). Based on the phylogenetic and the functional evidence using extant viruses as surrogates, we can attempt to infer the nature of these paleoviruses, although our “indirect paleovirology” is limited in its ability to formally prove the existence of such paleoviruses. Functional evidence that changes at residue 506 provide protection from antagonism by HCV is consistent with the hypothesis of ancient hepaciviruses being the causative agents of the evolution at residue 506. Since HCV per se is a human-specific virus, it is unlikely to have been responsible for MAVS evolution in non-human primates. Instead, we sought to find viruses with similar antagonistic properties as HCV. We found that NS3 proteases from GBV-A and GBV-C viruses, which naturally infect non-human primates, not only share the ability to antagonize MAVS but are also impeded by the same evolutionary changes at residue 506 (GBV-B protease also behaves in a similar manner to the other GB virus proteases and while it can experimentally infect some New World primates, its natural host is not known). These data suggest that despite the high degree of divergence, all hepaciviruses are capable of antagonizing MAVS. This shared property must have been present in their common ancestor and subsequently inherited by all extinct and present-day hepaciviruses. Furthermore, this common ancestor must have been present tens of millions of years ago. GBV-C viruses that infect humans and chimps are thought to have co-diverged with their ancestors 7 million years ago [47]. Similarly, GBV-A viruses are thought to have co-speciated with their hosts over the course of last 20 million years [47],[48]. Divergence of GBV-C from GBV-A and from their common ancestor from HCV presumably occurred even earlier. Taken together, these data suggest that hepaciviruses capable of antagonizing MAVS are an extremely ancient group of viruses that are old enough and distributed widely enough to be responsible for driving MAVS evolution at residue 506. Consistent with the ancient presence of hepaciviruses, we dated the change at residue 506 in the macaque lineage to 5–8 million years ago. Our findings imply that HCV and GB viruses represent the latest in a continuum of hepaciviral infections that have plagued primates for millions of years. Finally, it is interesting to note that MAVS in three Old World monkeys (OWM) has independently evolved resistance to hepaciviral antagonism despite the fact that hepaciviruses have not yet been detected in OWM.

Multiple lines of evidence outlined above support the hypothesis that ancient hepaciviruses were responsible for driving evolution in MAVS. However, it is also formally possible that a different class of viral antagonists unrelated to the hepaciviral NS3 proteases drove the evolution at residue 506. Although it is not formally possible to rule out such an alternative, there is little theoretical or empirical evidence in support of this. Residue 506 is not an “Achilles' heel” of MAVS that would make it an especially attractive target for antagonism by other viruses. There does not appear to be anything special about residue 506, except that it sits within a stretch of residues that corresponds to the cleavage site for hepaciviral protease NS3. Consistent with this idea, all other known viral antagonists of MAVS, including proteases from other viruses, interact with different regions of MAVS [9]–[12]. Furthermore, other residues under positive selection in MAVS are spread throughout the length of the protein, suggesting that MAVS can be antagonized along multiple surfaces and not just at or near residue 506. Taken together, these reasons make it unlikely that other viral antagonists besides hepaciviral proteases have converged to drive the evolution at residue 506.

### Inferring Paleovirology from Adaptive Evolution of MAVS

We have previously proposed that virus-driven adaptive evolution of host antiviral factors can be used as a tool in paleovirology, the study of ancient viruses and their impact of host evolution [49],[50]. The reasoning behind this idea is that positive selection in host antiviral factors essentially represents viral “footprints” that can reveal the action of ancient viruses. An inherent limitation of our “indirect” paleovirology approach is that it cannot formally rule out the possibility that either an unrelated virus or another selective pressure altogether was responsible for the observed evolution. Thus, in the case of evolution at residue 506 in MAVS, we cannot formally rule out alternate selection scenarios. The discovery of endogenized hepaciviral “fossils” in primate genomes might be an important confirmation of ancient hepaciviruses and their ability to infect primates. While such viral “fossils” would provide direct evidence for existence of paleoviruses, they would not necessarily reveal the functional consequences of these viruses on host evolution. Thus, “fossil”-based and “indirect” paleovirology should be viewed as complementary approaches, both of which are important to determine the identity of paleoviruses and their impact on host genomes.

One limitation of using adaptive evolution in host factors to study paleovirology that has been previously discussed [50] is the difficulty of deciphering the history of a single type of virus. For example, protein kinase R (PKR) is a broad-spectrum antiviral factor that is antagonized by a multitude of diverse viruses including ssRNA and dsDNA viruses, and often at the same domain. This is reflected in the large number of residues in PKR that are evolving under positive selection [18]. Such overlapping antagonism might obscure the history of individual viruses. Moreover, every subsequent round of adaptation might overwrite the paleoviral record of all previous infections. In contrast, despite MAVS acting against a broad panel of RNA viruses, there are only nine residues evolving under positive selection over primate evolution. This suggests that only a few viruses directly antagonize MAVS to drive its positive selection. This limited antagonism makes it less likely that more than one virus will converge on the same residue. Thus, studying proteins such as MAVS with sparse positive selection widely distributed along its length offers the opportunity to make paleoviral inferences with greater specificity.

Our study shows that the nature of the viral antagonist also contributes to the specificity of the paleoviral record. For example, it is surprising that the diverse hepaciviruses we tested have not dramatically altered the substrate specificity of their NS3 proteases over a long period of evolution. One explanation—supported by recent data from genomic viral “fossils” [51]—is that long-term virus evolution is much slower than expected due to functional constraint [50]. Indeed, the NS3 proteases are highly constrained because besides cleaving MAVS, they are also responsible for cleaving the viral polypeptide at multiple locations. Thus, given that paleovirology relies on the use of extant viruses as surrogates of their ancient counterparts to test the function of positively selected changes in host factors, viral “footprints” left by functionally constrained antagonists like proteases are particularly well suited for paleovirology [11],[12].

Functional dissection of the interaction between residue 506 in MAVS and HCV provides a model to study paleovirology using extant viruses and host immune factors. This model can be applied to the study of other residues in MAVS that have evolved under positive selection in primates, which we suspect also reflect a history of antagonism by other viruses. For example, coxsackievirus uses its 3C protease to cleave human MAVS between residues 148 and 149 (Figure S4) [12]. Remarkably, residue 149 has evolved under one of the strongest signatures of positive selection in MAVS (Figure 1B, Table S1). It is also interesting to note that residue 423, which forms the P5 position within the cleavage site for Hepatitis A virus protease, is also evolving under positive selection (Figure 1B, Table S1, Figure S4). Future experiments will be needed to establish the functional importance of residues 149 and 423. Nevertheless, the fact that the cleavage sites for all known protease antagonists of MAVS contain residues evolving under positive selection suggests that the correlation is unlikely to be simply a coincidence (Figure S4).

A particularly intriguing case of positive selection at residue 60 in MAVS also promises to reveal paleoviral insights (Figure 1B). This residue has independently acquired changes in six primates. In every instance, the ancestral asparagine has changed to serine (Figure S5). This selective change likely reflects the pressure to escape from some viral antagonism by evolving away from the ancestral asparagine, while continuing to maintain MAVS function, which might be particularly sensitive to changes at residue 60. Residue 60 occurs within the highly conserved CARD domain, which is utilized to directly interact with viral RNA sensors RIG-I and MDA-5 [6]. Interestingly, MAVS in colobus monkey is polymorphic for both asparagine and serine, suggesting that the selective pressure driving the change might be very recent. It will be interesting to functionally test whether serine at residue 60 provides protection from one of the known viral antagonists of MAVS. Alternatively, the dramatic convergent evolution at residue 60 might help discover a new viral antagonist of MAVS.

## Materials and Methods

### Sequencing MAVS cDNA

Isolation of total RNA was done as described previously [18]. Primate MAVS cDNAs were isolated by RT-PCR using 50 ng of total RNA as template and TA-cloned into pCR2.1 (Invitrogen). Primer MP-11, which sits upstream of the start site, and primer MP-12, which is downstream of the stop codon, were used for RT-PCR (Table S2). MP-34, along with MP-11 and MP-12, was used for sequencing the cDNAs. MAVS cDNA sequences obtained in this study are in the process of being submitted to GenBank. Common marmoset sequence was obtained from the UCSC Genome Browser.

### Plasmid Construction

Primate MAVS cDNAs were prepared using 250 ng total RNA as template and oligo dT primer (Superscript III, Invitrogen). cDNAs were subsequently used as a template for PCR (Phusion, NEB). MAVS cDNAs were cloned into pFLAG-CMV-2 between Hind*III* and Bgl*II* downstream of and in frame with FLAG tag.

Single amino acid changing point mutations were introduced in human MAVS as follows: MP-51, a forward primer upstream of the MAVS cDNA in pFLAG-CMV-2, was used in combination with a reverse primer that included the desired change. A forward primer with the desired change was then used with MP-52, a reverse primer downstream of MAVS cDNA in pFLAG-CMV-2. The resulting products from these two PCRs were used as a template for stitch PCR with MP-51 and MP-52 primers. The resulting PCR product, which included the desired change in MAVS, was ligated into pFLAG-CMV-2 between Hind*III* and Bgl*II* in frame with FLAG tag. The remaining single amino acid changes in MAVS were introduced using similar stitching strategy, but desired amino acid changes were achieved by stitching together N- and C-terminal MAVS of different but closely related species.

NS3/4A from GBV-B, GBV-C, HCV, and GBV-A was cloned into pcDNA3.1/Hygro(−) between Xba*I* and Hind*III* downstream of and in frame with HA tag (cloned between Nhe*I* and Xba*I*).

GBV-A NS3/4A was synthesized (Genscript Inc., Piscataway, New Jersey) using strain A-lab sequence.

Other plasmids used in this study were: pJC453–IFN-β promoter luciferase firefly reporter construct (kindly provided by Zhijian Chen), pRL-TK–renilla transfection control construct (Promega Inc.), pcDNA6-BVDVpro (kindly provided by Kui Li), pcDNA6-YFVpro (kindly provided by Kui Li), Human MAVS (C508Y) (previously described [52]), and pcDNA-Myc-sc-NS3 (S1165A) (previously described [33]).

### Evolutionary Analyses

Amino acid sequence alignment was performed using ClustalW. This amino acid alignment was used as input for DNA sequence alignment in PAL2NAL. Aligned DNA sequences were subsequently used for all analyses done in PAML software. The well-supported primate phylogeny (Figure 1A) [26] was used for analyses done in PAML [28]. Maximum-likelihood analysis to determine whether MAVS is evolving under positive selection and to determine which residues are evolving under positive selection was done using the sites models in codeml program of PAML [28]. Sites models allow the dN/dS ratio to vary among residues. Branch models, which allow the dN/dS ratio to vary among different branches of the phylogeny, were used to determine dN/dS values for different lineages. Two-ratio tests to detect episodic selection were performed by comparing a likelihood model that allows dN/dS to vary across phylogeny to model with dN/dS fixed at 1. REL and branch-site REL analysis were performed using a web-based implementation of HyPhy package (www.datamonkey.org). Branch-site REL is based on likelihood ratio tests that identify all lineages with a proportion of sites that are evolving with dN/dS>1, without making a priori assumptions about which lineages these are in the phylogeny [29].

### Luciferase Assays

5×10^4^ HEK293 cells were plated per well in 96-well plates and transfected on the next day with LT1 (Mirus) transfection reagent. The following plasmids were co-transfected in triplicates: MAVS plasmid (2.5 ng), luciferase reporter construct pCJ453 (50 ng), transfection control pRL-TK construct (10 ng), none or one of the viral protease encoding plasmids (pJC1461—2.5 ng, pMP125—2.5 ng, pMP122—10 ng, pMP123—2.5 ng, pMP126—10 ng), and pFLAG-CMV-2 (enough to bring final DNA concentration equal to 150 ng). One triplicate set of wells was transfected without MAVS plasmid and one set without any DNA. 24 h after transfection, Dual-Glo Luciferase Assay System (Promega) was used to lyse the cells and measure luciferase firefly and renilla activity with a luminometer. Amount of firefly or renilla activity in wells transfected without any DNA was subtracted as background. Firefly activity was divided by the renilla activity in the same well to control for transfection efficiency. This firefly/renilla ratio from cells expressing MAVS was divided by the firefly/renilla ratio from wells without MAVS to calculate IFN-β fold induction, which was then averaged across the set of three wells.

### Immunoblot Analysis to Assay Cleavage

It was not possible to get enough lysate from a single well of a 96-well plate for immunoblot analysis. We therefore scaled the entire transfection process by 4-fold in 24-well plates. Therefore, 20×10^5^ cells were plated per well in 96-well plates and transfected with 4 times as much LT1 transfection reagent. Concentration of all the plasmids was also increased 4-fold. 24 h after transfection, cells were resuspended in 36 µl NTE buffer (10 mM Tris—pH 8, 1 mM EDTA, 50 mM NaCl) containing 0.18 mg protease inhibitor cocktail Complete Mini (Roche). Cells were then lysed on ice for 10 min in 44 µl of NP-40 buffer (1% NP-40, 0.2% sodium-deoxycholate, 0.12 M NaCl, 20 mM Tris—pH 8) containing 0.22 mg protease inhibitor cocktail, 2.4×10^−6^ mol DTT, and 1.2×10^−6^ mol PMSF. Lysate was spun at maximum speed on tabletop centrifuge for 10 min. 75 µl of supernatant was mixed with 19 µl of 5× SDS sample buffer with 10% β-ME. Samples were boiled for 10 min and then 5–10 µl was used for Western blot analysis.

Mouse monoclonal anti-FLAG M2 antibody (Sigma-Aldrich) was used to detect FLAG-tagged MAVS, and mouse monoclonal anti-HA.11 antibody (Covance) was used to detect HA-tagged HCV NS3/4A. Rabbit polyclonal anti-beta Actin antibody (Abcam) was used to detect actin. HRP-linked anti-mouse and anti-rabbit IgG antibodies (GE Healthcare) were used as secondary anti-bodies. Primary anti-bodies were used at 1∶1,000 dilution at 4° overnight and secondary anti-bodies were used at 1∶10,000 dilution for 1 h at room temperature. SuperSignal West Dura ECL substrate (Thermo Scientific) was used for detecting HRP.

### Co-Immunoprecipitation Assay

For co-immunoprecipitation analysis, HEK 293 cells were transfected with empty vector or pcDNA-Myc-sc-NS3 (S1165A) protease [33] that contains aa 21–32 of HCV NS4A fused to aa 1026–1206 of the HCV NS3 protease with the protease active site mutation S1165A, as well as empty vector or MAVS-expressing constructs. Cells were lysed in 1% Triton X-100, 150 mm NaCl, and 25 mM Tris-Cl pH 7.5. Coimmunoprecipitation was performed using FLAG M2-agarose beads (Sigma-Aldrich) followed by immunoblot analysis using the following antibodies: anti-myc (Abcam), anti-Flag M2-Peroxidase (Sigma-Aldrich), and anti-tubulin (Sigma-Aldrich).

### HCV Restriction Assay

Huh7-K2040 is a Huh7-based human hepatocyte cell line that contains the self-replicating subgenomic replicon from HCV 1b [34]. Huh7-K2040 is transfected with control vector or plasmid expressing the indicated MAVS construct for 36 h. Cell lysates were analyzed by immunoblot with antiserum specific to HCV NS5A [53], tubulin (Sigma-Aldrich), and FLAG M2 (Sigma-Aldrich) for detection of the various MAVS constructs. HCV protein was further detected using the hyperimmune serum from an HCV-infected patient that recognizes HCV proteins NS3, NS4B, and NS5A [35]. Protein densitometry was determined using the NIH ImageJ program. To derive fold restriction, NS5A (rabbit polyclonal)/tubulin ratio for each sample was divided by the NS5A(rabbit polyclonal)/tubulin ratio from the vector alone control.

## Supporting Information

Figure S1 Schematic of the RIG-I/MDA-5 pathway. Solid arrows indicate direct interaction. Through a signaling cascade, MAVS activates IRF-3 and NFκ-B, which bind to and activate IFN-β promoter.
(TIF)

Figure S2 Ability of MAVS from multiple primate species to induce IFN-β activity. Same data as in Figure 2, but fold IFN-β induction is not normalized. Induction of IFN-β promoter, as measured by luciferase firefly activity, upon expression of MAVS cDNA from corresponding species coexpressed with (+) or without (−) HCV NS3/4A. Primates with MAVS capable of significant IFN-β induction even in presence of HCV protease are highlighted in bold. Human (C508R) refers to substitution of Cysteine (C) at position 508 with Arginine (R) in human MAVS. All experiments are done in triplicates, and error bars indicate standard deviation.
(TIF)

Figure S3 Residue 506 provides protection against NS3/4A from all hepaciviruses. Same data as in Figure 6 but includes data from control groups (IFN-β induction in absence of any protease). IFN-β induction due to expression of MAVS from indicated species, as determined by luciferase firefly activity, is presented for either the ancestral “susceptible” valine or derived “resistant” variants (in red) at position 506 as shown. Luciferase firefly activity is normalized as being 100% in absence of the protease. Susceptibility to each of the GB-virus protease is shown. *y*-axis is the same for all graphs. All experiments done in triplicates. Error bars indicate standard deviation.
(TIF)

Figure S4 Positive selection within the viral protease cleavage sites. Cleavage sites in MAVS of three viral proteases have been mapped so far (indicated by the scissors). Residues between which proteases cleave MAVS are indicated. Note the presence of residues under positive selection (triangles) within or proximal to the cleavage sites of all three proteases.
(TIF)

Figure S5 MAVS protein alignment. Residues evolving under positive selection are highlighted in yellow.
(PDF)

Table S1 Statistical evidence for sites evolving under positive selection in MAVS. Likelihood ratio tests for positive selection in MAVS were done using NSsites model M7 versus M8 comparison assuming F61 model of codon frequency in codeml program in PAML software. Likelihood ratio tests were also performed using random effects likelihood (REL), implemented in web-based HyPhy package. Residues (highlighted in bold) with Bayes Empirical Bayes (BEB) and REL calculated posterior probabilities (PPr) equal to or greater than 0.9 for which dN/dS>1 are shown in Figure 1B. NEB, Naive Empirical Bayes.
(TIF)

Table S2 List of primers used in the study. Primers are in 5′ to 3′ orientation.
(TIF)

## Abbreviations

BEB: Bayes Empirical Bayes
BVDV: bovine viral diarrhea virus
CARDIF: CARD adapter inducing interferon-beta
dN: nonsynonymous
dS: synonymous
HCV: Hepatitis C Virus
IPS-1: interferon beta promoter stimulator protein 1
MAVS: mitochondrial antiviral signaling protein
NS3: non-structural protein 3
NS4A: non-structural protein 4A
OWM: Old World monkeys
PKR: Protein Kinase R
PPr: posterior probabilities
REL: random effects likelihood
VISA: virus-induced-signaling adapter
YFV: yellow fever virus
