# Supplementary figures and images for: Convergent Evolution of Escape from Hepaciviral Antagonism in Primates
Source: PLoS Biol. 2012 Mar 13;10(3):e1001282. doi: 10.1371/journal.pbio.1001282 (PMC3302847; doi:10.1371/journal.pbio.1001282)

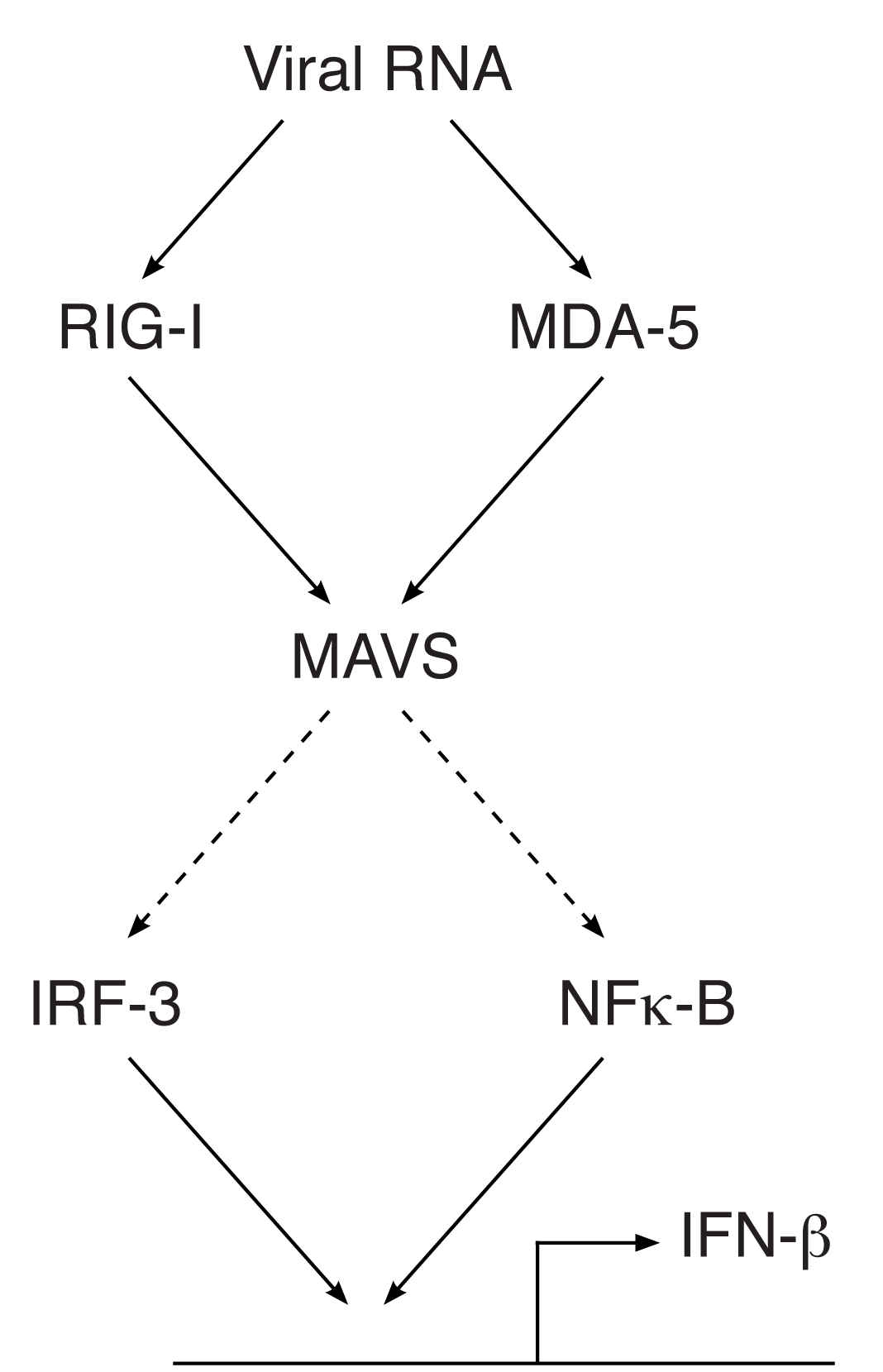

Supplement: Figure S1 — Schematic of the RIG-I/MDA-5 pathway. Solid arrows indicate direct interaction. Through a signaling cascade, MAVS activates IRF-3 and NFκ-B, which bind to and activate IFN-β promoter. (TIF) [file pbio.1001282.s001.tif]

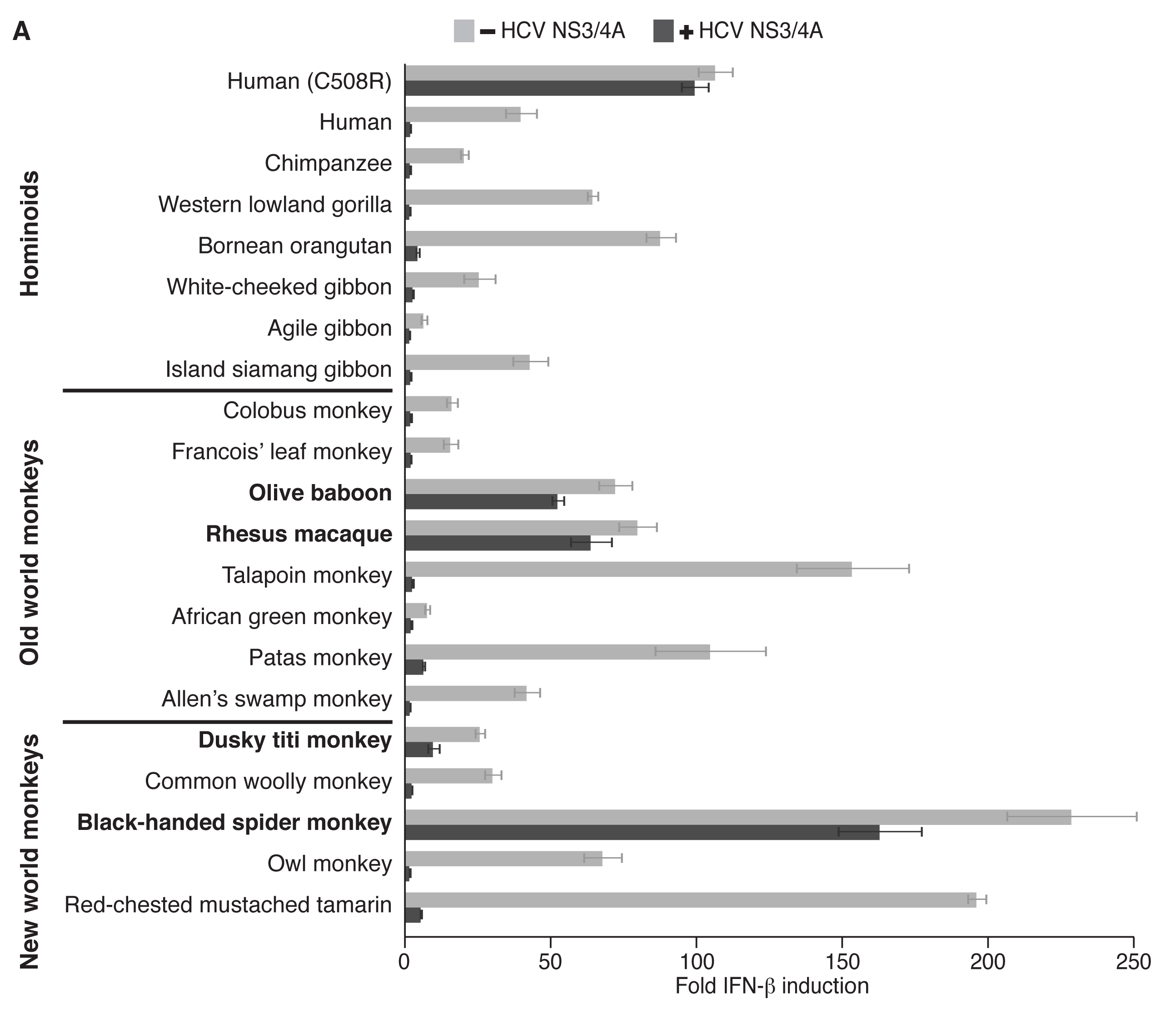

Supplement: Figure S2 — Ability of MAVS from multiple primate species to induce IFN-β activity. Same data as in Figure 2, but fold IFN-β induction is not normalized. Induction of IFN-β promoter, as measured by luciferase firefly activity, upon expression of MAVS cDNA from corresponding species coexpressed with (+) or without (−) HCV NS3/4A. Primates with MAVS capable of significant IFN-β induction even in presence of HCV protease are highlighted in bold. Human (C508R) refers to substitution of Cysteine (C) at position 508 with Arginine (R) in human MAVS. All experiments are done in triplicates, and error bars indicate standard deviation. (TIF) [file pbio.1001282.s002.tif]

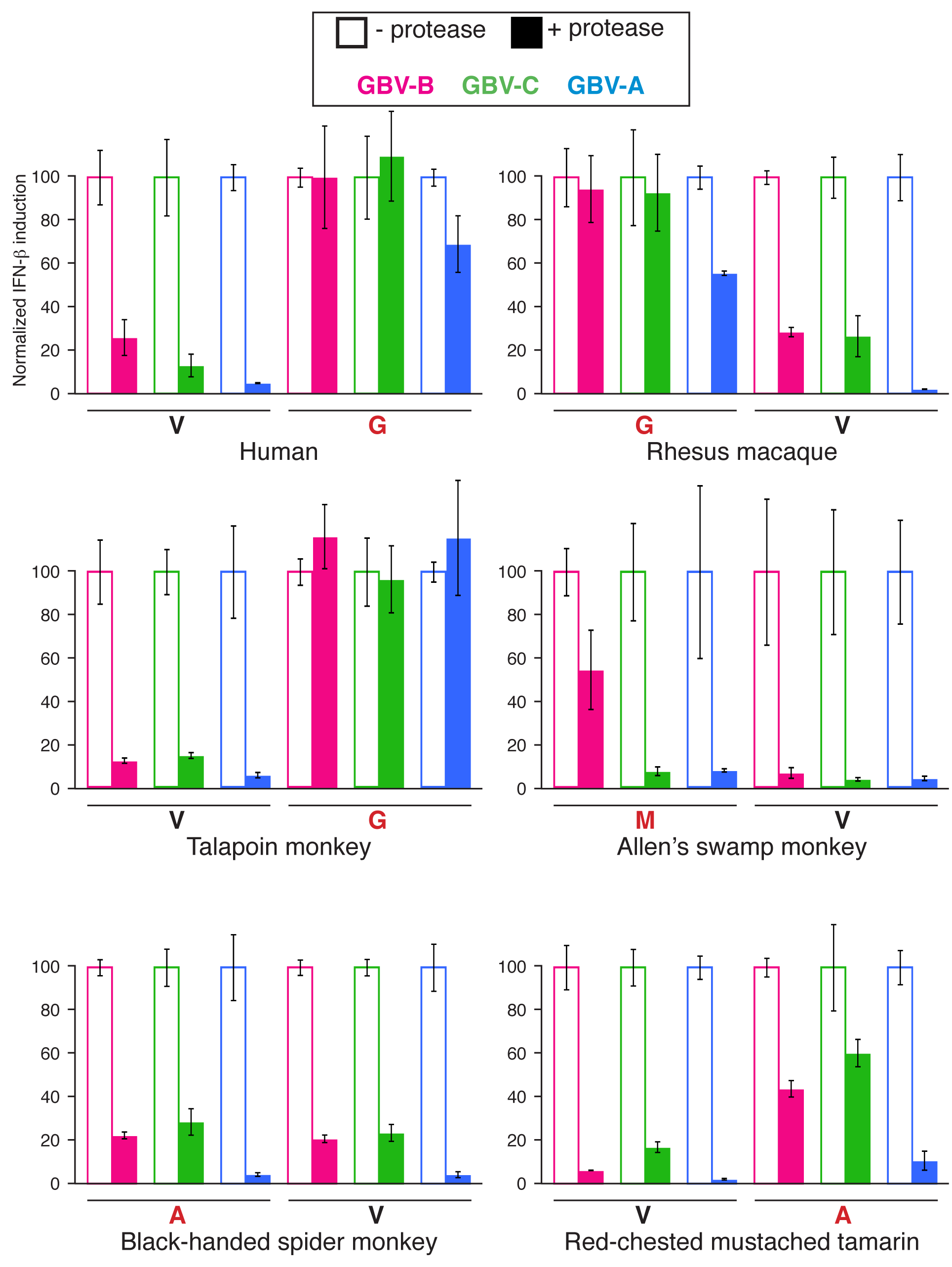

Supplement: Figure S3 — Residue 506 provides protection against NS3/4A from all hepaciviruses. Same data as in Figure 6 but includes data from control groups (IFN-β induction in absence of any protease). IFN-β induction due to expression of MAVS from indicated species, as determined by luciferase firefly activity, is presented for either the ancestral “susceptible” valine or derived “resistant” variants (in red) at position 506 as shown. Luciferase firefly activity is normalized as being 100% in absence of the protease. Susceptibility to each of the GB-virus protease is shown. y-axis is the same for all graphs. All experiments done in triplicates. Error bars indicate standard deviation. (TIF) [file pbio.1001282.s003.tif]

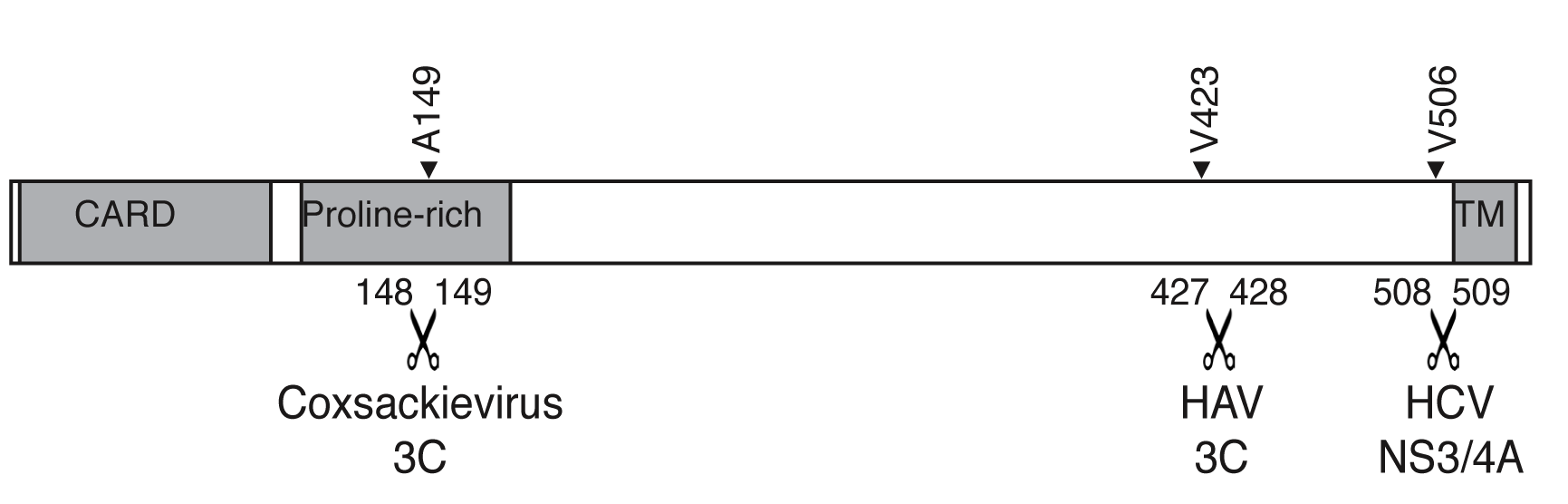

Supplement: Figure S4 — Positive selection within the viral protease cleavage sites. Cleavage sites in MAVS of three viral proteases have been mapped so far (indicated by the scissors). Residues between which proteases cleave MAVS are indicated. Note the presence of residues under positive selection (triangles) within or proximal to the cleavage sites of all three proteases. (TIF) [file pbio.1001282.s004.tif]

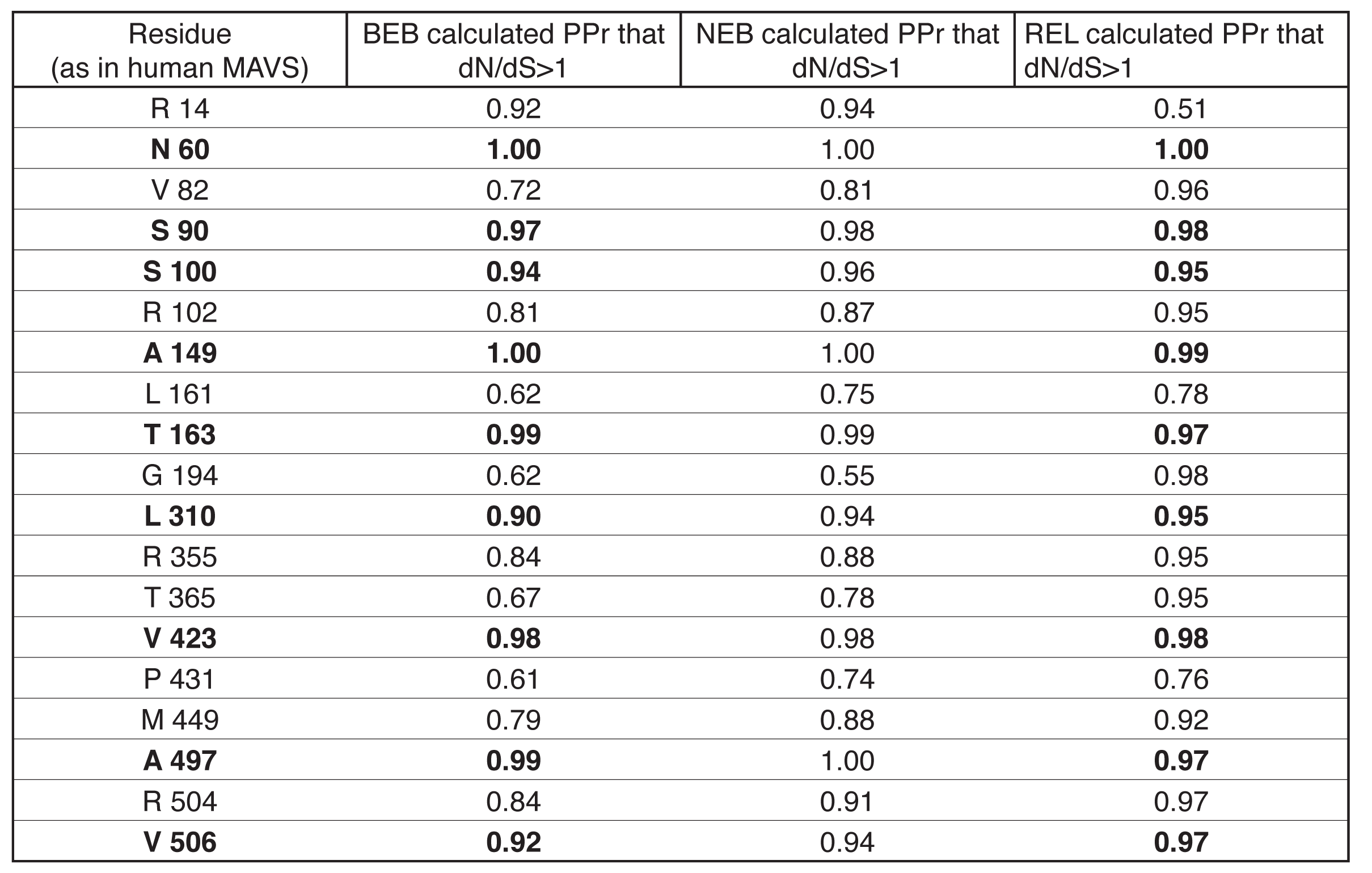

Supplement: Table S1 — Statistical evidence for sites evolving under positive selection in MAVS. Likelihood ratio tests for positive selection in MAVS were done using NSsites model M7 versus M8 comparison assuming F61 model of codon frequency in codeml program in PAML software. Likelihood ratio tests were also performed using random effects likelihood (REL), implemented in web-based HyPhy package. Residues (highlighted in bold) with Bayes Empirical Bayes (BEB) and REL calculated posterior probabilities (PPr) equal to or greater than 0.9 for which dN/dS>1 are shown in Figure 1B. NEB, Naive Empirical Bayes. (TIF) [file pbio.1001282.s006.tif]

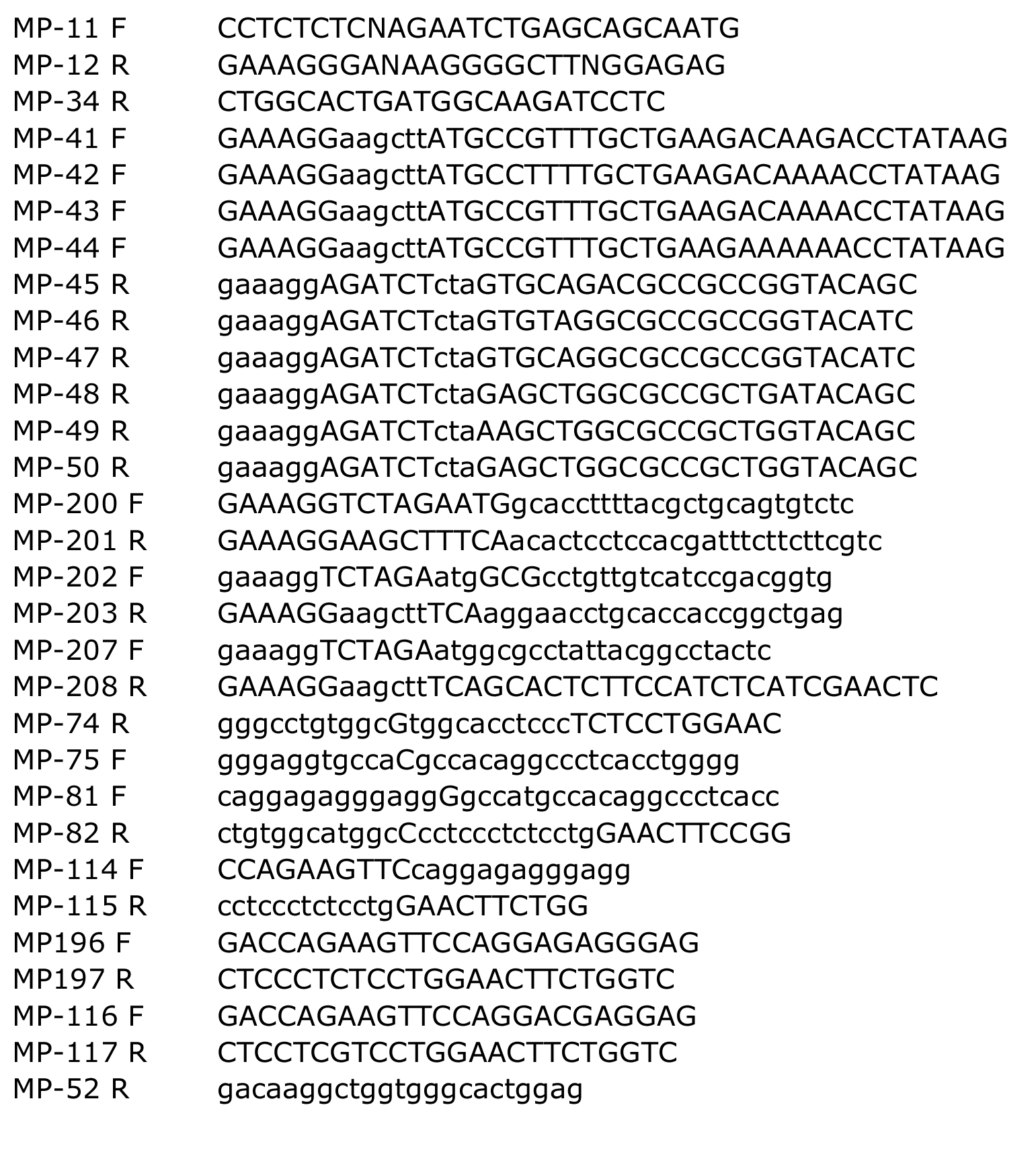

Supplement: Table S2 — List of primers used in the study. Primers are in 5′ to 3′ orientation. (TIF) [file pbio.1001282.s007.tif]
