# Supplementary material for: Convergent Evolution of Escape from Hepaciviral Antagonism in Primates
Source: PLoS Biol. 2012 Mar 13;10(3):e1001282. doi: 10.1371/journal.pbio.1001282 (PMC3302847; doi:10.1371/journal.pbio.1001282)
Supplement: Figure S5 — MAVS protein alignment. Residues evolving under positive selection are highlighted in yellow. (PDF) [file pbio.1001282.s005.pdf]

```
Human                          MPFAEDKTYKYICRNFSNFCNVDVVEILPYLPCLTARDQDRLRATCTLSG 50
Chimpanzee                     MPFAEDKTYKYICRNFSNFCNVDVVEILPYLPCLTARDQDRLRATCTLSG 50
Western_lowland_gorilla        MPFAEDKTYKYICRNFSNFCNVDVVEILPYLPCLTARDQDRLRATCTLSG 50
Bornean_orangutan              MPFAEDKTYKYICHNFSNFCNVDVVEILPYLPCLTARDQDRLRATCTLSG 50
White-cheeked_gibbon           MPFAEDKTYKYICHNFSNFCNVDVVEILPYLPCLTARDQDRLRATCTLSG 50
Agile_gibbon                   MPFAEDKTYKYICHNFSNFCNVDVVEILPYLPCLTARDQDRLRATCTLSG 50
Island_siamang_gibbon          MPFAEDKTYKYICHNFSNFCNVDVVEILPYLPCLTARDQDRLRATCTLSG 50
Colobus_monkey                 MPFAEDKTYKYICRNFSNFCNVDVVEILPYLPCLTARDQDRLRATCTLSG 50
Francois'_leaf_monkey          MPFAEDKTYKYICRNFSNFCNVDVVEILPYLPCLTARDQDRLRATCTLSG 50
Olive_baboon                   MPFAEDKTYKYICRNFSNFCNVDVVEILPYLPCLTARDQDRLRATCTLSG 50
Rhesus_macaque                 MPFAEDKTYKYICRNFSNFCNVDVVEILPYLPCLTARDQDRLRATCTLSG 50
Talapoin_monkey                MPFAEDKTYKYICRNFSNFCNVDVVEILPYLPCLTARDQDRLRATCTLSG 50
African_green_monkey           MPFAEDKTYKYICRNFSNFCNVDVVEILPYLPCLTARDQDRLRATCTLSG 50
Patas_monkey                   MPFAEDKTYKYICRNFSNFCNVDVVEILPYLPCLTARDQDRLRATCTLSG 50
Allen's_swamp_monkey           MPFAEDKTYKYICRNFSNFCNVDVVEILPYLPCLTARDQDRLRATCTLSG 50
Duski_titi_monkey              MPFAEDKTYKYICRHFSNFCNVDVVEILPYLPCLTARDQDRLRATCTLSG 50
Common_woolly_monkey           MPFAEDKTYKYICRHFSNFCNVDVVEILPYLPCLTARDQDRLRAICTLSG 50
Black-handed_spider_monkey     MPFAEDKTYNYICRHFSNFCNVDVVEILPYLPCLTARDQDRLRAICTLSG 50
Owl_monkey                     MPFAEDKTYKYICRHFSNFCNVDVVEILPYLPCLTARDQDRLRATCTLFG 50
Red-chested_mustached_monkey   MPFAEEKTYKYICRHFSNFCNVDVVEILPYLPCLTARDQDRLRATCTLSG 50
Common_marmoset                MPFAEDKTYKYICRHFSNFCNVDVVEILPYLPCLTARDQDRLRATCTLSG 50
                               *****:***:***::***************************** *** *

Human                          NRDTLWHLFNTLQRRPGWVEYFIAALRGCELVDLADEVASVYQSYQPRTS 100
Chimpanzee                     NRDTLWHLFNTLQRRPGWVEYFIAALRGCELVDLADEVASVYQSYQPRTS 100
Western_lowland_gorilla        NRDTLWHLFSTLQRRPGWVEYFIAALRDCELVDLADEVASVYQSYQPRTS 100
Bornean_orangutan              NRDTLWYLFNTLQRRPGWVEYFIAALRGCELSDLADEVACVYQSYQPRTL 100
White-cheeked_gibbon           NRDTLWHLFNTLQRRPGWVEYFITALRGCELADLADEVACVYQSYQPRTL 100
Agile_gibbon                   NRDTLWHLFNTLQRRPGWVEYFITALRGCELADLADEVACVYQSYQPRTL 100
Island_siamang_gibbon          NRDTLWHLFNTLQRRPGWVEYFITALRGCELADLADEVACVYQSYQPRTL 100
Colobus_monkey                 NRDTLWHLFSTLQRRPGWVESFIAALRDCELADLADEVACVYQRYQPRTS 100
Francois'_leaf_monkey          NRDTLWHLFNTLQRRPGWVESFIAALRDCELADLADEVAGVYQSYQPRTS 100
Olive_baboon                   NRDTLWHLFNTLQRRPGWVESFIAALRDCELADLADEVACVYQSYQPRTL 100
Rhesus_macaque                 NRDTLWHLFSTLQRRPGWVESFIAALRDCELADLADEVACVYQSYQPRTS 100
Talapoin_monkey                NRDTLWHLFNTLQRRPGWVESFIAALRDCELADLADEVACVYQSYQPRTS 100
African_green_monkey           NRDTLWHLFSTLQRRPGWVESFIAALRDCELADLADEVACVYQSYQPRTS 100
Patas_monkey                   NRDTLWHLFNTLQRRPGWVESFIAALRDCELADLADEVACVYQSYQPRTS 100
Allen's_swamp_monkey           NRDTLWHLFNTLQRRPGWVESFIAALRDCELADLADEVACVYQSYQPRTS 100
Duski_titi_monkey              NRDTLWHLFNTLQRRPGWVESFIVALRACELSDLADEVARVYQSYQPRTP 100
Common_woolly_monkey           NRDTLWHLFSTLQRRPGWVESFVVALRACELSDLADEVARVYQSYQPRTP 100
Black-handed_spider_monkey     NRDTLWHLFNTLQRRPGWVESFTVALRACELSDLADEVACAYQSYQPRTP 100
Owl_monkey                     NRDTLWHLFSTLQRRPGWVESFIVALRACELSELADEVTRVYQSYQPRTP 100
Red-chested_mustached_monkey   NRDTLWHLFNALQRRPGWVESFIVALRACELSDLADEVARVYQSYQPRTP 100
Common_marmoset                NRDTLWHLFNTLQRRPGWVESFIVALRACELSDLADEVARVYQSYQPRTP 100
                               ******:**.:********* * .*** *** :*****: .** ***** 
```

```
Human                            DRPPDPLEPPSLPAERPGPPTPAAAHSIPYNSCREKEPSYPMPVQETQAP 150
Chimpanzee                       DCPPDPLEPPSLPAERPGPPTPAAAHSIPYNSCREKEPSYPMPVQETQVP 150
Western_lowland_gorilla          DRPPDPLEPPSLPAERPGPPTPAAAHSIPYNSCREKEPSYPMPVQETQAP 150
Bornean_orangutan                DRPPDPLEPPSLPAEGPGPPTPAAAHSIPYNGCREKEPSYLMPVQETQAP 150
White-cheeked_gibbon             DRPPDPLEPPSLPAEGPGPPTPAAAHSIPYNGCREKEPSYPMPVQETQVP 150
Agile_gibbon                     DRPPDPLEPPSLPAEGPGPPTPAAAHSIPYNGCREKEPSYPMPVQETQVP 150
Island_siamang_gibbon            DRPPDPLEPPSLPAEGPGPPTPAAAHSMPYNGCREKEPSYPMPVQETQVP 150
Colobus_monkey                   DRPLDPLEPPSLPAEGPGPPTPAVAHSIPYNGYREKEPSYPMSVQETQAP 150
Francois'_leaf_monkey            DRPLDPLEPPSLPAEGPGPPTPAAAHSIPYNGYREKEPSYPMPVQETQVP 150
Olive_baboon                     DRPPDPLEPPSLPAEGPGPPTPAAAHSIPYNGYREKEPSYPMPVQETQAP 150
Rhesus_macaque                   DRPPDPLEPPSLPAEGPGPPTPAATHSIPYNGYREKEPSYPMPVQETQAP 150
Talapoin_monkey                  DRPPDPLEPPSLPAEGPGPPTPAAAHSIPYNGYREKEPSYPMPVQETRAP 150
African_green_monkey             DRPPDPLEPPSLPAEGPGPPTPAAAHSIPYNSYREKEPSYPMPVQETRAP 150
Patas_monkey                     DRPPDPLEPPSLPAEGPGPPTPAAAHSIPYNGYREKEPSYPMPVQETRAP 150
Allen's_swamp_monkey             DRPPDPLEPPSLPAEGPGPPTPAAAYSIPYNGYREKEPSYPMPVQETRAP 150
Duski_titi_monkey                EYPPAPLEPPSVSAEVPGPSTPAVAYSIPYNGYREKEPSYPMPVQETQLP 150
Common_woolly_monkey             EHPPAPLEPPSVSAEVQGPSTPAAAHSIPYNGYREKEPSYPVPVQETQLP 150
Black-handed_spider_monkey       EHPPAPLEPPSVSAEVPGPSTPAAAHSIPYNGYREKEPSYPMPVQETQLP 150
Owl_monkey                       EHPSAPLEPPSVSAEVPGPSTPAAAHSIPYNGYREKEPSYPMPVQDTQPP 150
Red-chested_mustached_monkey     EHPPAPLEPPSVSAEVPGPSTPAAAHSIPYNGYREKEPSYPMPVQESQPP 150
Common_marmoset                  EHPPAPLEPPSVSAEVPGPSTPAAAHSIPYNGYREKEPSYPMPVQESQPP 150
                                 : *  ******:.**  **.***.::*:***. ******* :.**::: *

Human                            ESPGENSEQALQTLSPRAIPRNPDGGPLESSSDLAALSPLTSSGHQEQDT 200
Chimpanzee                       ESPGESSEQALQTLSPRAIPRNPDGGPLESSSDLAALSPLTSSGHQEQDT 200
Western_lowland_gorilla          ESPGESSEQALRMLSPRAIPRSPDGGPLESSSDLAALSPLTSSGHQEQDT 200
Bornean_orangutan                ESPGESSEQARQMLSPRAIPRSPDGGPLESSSDLAALSPLTSSGHQEQDT 200
White-cheeked_gibbon             ESPGESSEQAPQMLSPRAIPRSPDGGPLESSSDLAALSPLTSSGHQEQDR 200
Agile_gibbon                     ESPGESSEQAPQMLSRRAIPRSPDGGPLESSSDLAALSPLTSSGHQEQDR 200
Island_siamang_gibbon            ESPGESSEQAPQMLSPRAIPRSPDGGPLESSSDLAALSPLTSSGHQEQDR 200
Colobus_monkey                   ESPGESSEQAPQTLNPRVIPRSPDGGPLEPSSD---LSPLTSSGHQEQDT 197
Francois'_leaf_monkey            ESPGESSEQAPQTLNPRVIPRSPDGGPLEPSSDLAALSPLTSSGHQEQDT 200
Olive_baboon                     DSPGESSEQAPQTLNPRVIPRSPDGGPLEPSSDLAALSPLTSSGHQEQDT 200
Rhesus_macaque                   DSPGESSEQAPQTLNPRVIPRSADGGPLEPSSDLAALSPLTSSGHQEQDT 200
Talapoin_monkey                  DSPGESSEQAPQTLNPRVIPRSPDGGPLEPSSDLAALSPLTSSGHQEQDT 200
African_green_monkey             DSPGESSEQAPQILNPRVIPRSPDGGPLDPSSDLAALSPLTSSGHQEQDT 200
Patas_monkey                     DSPGESSEQAPQTLNPRVIPRSPDGGPLDPSSDLAALSPLTSSEHQEQDT 200
Allen's_swamp_monkey             DSPGESSEQAPQTLNPRVIPRSPDGGPLEPSSDLAALSPLTSSRHQEQDT 200
Duski_titi_monkey                ESLEESSEQAPQMLSPGAVPRRPVGGPVEPSSDLAALSPLTSSRHQEQDT 200
Common_woolly_monkey             ESLEESSEQAPQTLSPGAIPRRPGGGPVEPSSDLAALSPLTSSRHQEQDT 200
Black-handed_spider_monkey       ESLEESSEQAPQTLSPGAIPRRPGGSPVEPSSDLAAFSPVTSSRHQEQDT 200
Owl_monkey                       ETLEESSEQAPQTLSPGAIPRRPGGSPVEPSSDLAALSPLTSSGHQEQDT 200
Red-chested_mustached_monkey     ESLEESSEQAAQTLSPGAIPRRPGGGPVEPSSDLAALSPVTSSGHQEQDT 200
Common_marmoset                  ESLEESSEQAPQMLSPGAIPRRLGGGPVEPSADLAALSPVTSSGHQEQDT 200
                                 ::  *.**** : *.  .:**   *.*::.*:*   :**:*** *****
```

```
Human                            ELGSTHTAGATSSLTPSRGPVSPSVSFQPLARSTPRASRLPGPTGSVVST 250
Chimpanzee                       ELGSTHTAGATSSLTPSRGPVSPSVSFQPLARSTPRASHLPGPTVSVVST 250
Western_lowland_gorilla          ELGSTHTAGATSSLTPSRGPVSPSVSFQPLARSTPRASRLPGPTGSVVST 250
Bornean_orangutan                ELGSTHIAGATSSLTPSRGPVSPSVSFQPLARSTPRASRLPGPTGSVLST 250
White-cheeked_gibbon             ELGSTHTAGATSSLTASRGPVSPSVSFQPLARSTPRASRLPGPTGSVVSP 250
Agile_gibbon                     ELGSTHTAGATSSLTPSRGPVSPSVSFQPLARSTPRASRLPGPTGSVVSP 250
Island_siamang_gibbon            ELGSTHTAGATSSLTPSRGPVSPSVSFQPLARSTPRASRLPGPTGSVVSP 250
Colobus_monkey                   ELGSTHTAGVTSSLTPSRGPVSPSVSFQPLARSTPRASRLPGPAGSVVST 247
Francois'_leaf_monkey            ELGSTHTADATSSLTPSRGPVSPSVSFQPLARSTPRASRLPGPAGSVVST 250
Olive_baboon                     ELGSTHTKGATSSLIPSRGPVSPSVSFQPLARSTPRASRLPGHAGSVVST 250
Rhesus_macaque                   ELGSTHTAGATSSLIPSRGPVSPSVSFQPLARSTPRASRLPGPAGSVVST 250
Talapoin_monkey                  ELGSTHTAGATSSLTPSRGPVSPSVSFQPLARSTPRASRLPGPAGSVVST 250
African_green_monkey             ELGSTHTAGATSSLTPSRGPVSPSVSFQPLARSTPRASRLPGPAGSVVST 250
Patas_monkey                     ELGSTHTAGATSSLTPSRGPVSPSVSFQPLARSTPRASRLPGPAGSVVST 250
Allen's_swamp_monkey             ELGSTHTAGATSSLTPSRGPVSPSVSFQPLARSTPRASRLPGPAGSVVST 250
Duski_titi_monkey                ELGSTHTAGATSSLTPSRGPVSPSVSFQPLARSTPRASRLPGPAGS-VST 249
Common_woolly_monkey             ELGSTHTAGAPSSLTPSRGPVSPSVSFQPLPRSTPRKSRLPGPAGS-VST 249
Black-handed_spider_monkey       ELGSTHTAGAPSSLTPSRGPVSPSVSFQPLPRSTPRKSRLPGPAGS-VST 249
Owl_monkey                       ELGSTHTAGATTSLTPSRGPVSPSVSFQPLSRSTPRASRLRGPAGS-VST 249
Red-chested_mustached_monkey     ELGSTHIAGATSSLTPSRGPVSPSVSFQPLARSTPRASRLPGPAGS-VST 249
Common_marmoset                  ELGSTHTAGATSSLTPSRGPVSPSVSFQPLARSTPRASRLPGPAGS-VST 249
                                 ******  ...:** .**************.***** *:* * : * :*.

Human                            GTSFSS--SSPGLASAGAAEGKQGAESDQAEPIICSSGAEAPANSLPSKV 298
Chimpanzee                       GTSFSS--SSPGLASAGAAEGKQGAESDQAEPIICSSGAEAPANSLPSKV 298
Western_lowland_gorilla          GTSFSS--SSPGLASAGAAEGKQGAESDQAEPIICSSGAEAPANSLPSKV 298
Bornean_orangutan                GTSSSS--SSPGLASAGAAEGKQGAESDQAEPITCSIGAEAPANSRPSKV 298
White-cheeked_gibbon             GTSSSS--SSPGLASAGAAESKQGAESDQAEPIICSRGAEAPANSLPSKV 298
Agile_gibbon                     GTSSSS--SSPGLASAGAAEGKQGAESDQAEPIICSSGAEAPANSLPSKV 298
Island_siamang_gibbon            GTSSSS--SSPGLASAGAAEGKQGAESDQAEPIICSSGAEAPANSLPSKV 298
Colobus_monkey                   GTSSSS--S-PGLASAGAAEGEEGAESDQAEPIICSSGAEAPANSLPSKV 294
Francois'_leaf_monkey            GTSSSS--SSPGLASAGAAEGEQGAESDEAEPIICSSGAEAPANSLPSKV 298
Olive_baboon                     GTSSSSSSSSPGLASAGAAEGDQGAESDQAEPIICSSGAEAPANSLPSKV 300
Rhesus_macaque                   GTSSSSSSSSPGLASAGAAEGEQGAESDQAEPIICSSGAEAPANSLPSKV 300
Talapoin_monkey                  GTSSSSS-SSPGLASAGAAEGEQGAESDQAEPIICSSGAEAPANSLPSKV 299
African_green_monkey             GTSSSSS-SSPGLASAGAAEGEQGAESDQAEPIICSSGAEAPANSLPSKV 299
Patas_monkey                     GTSSSSS-SSPGLASAGAAEGEQGAESDQAEPIICSSGAEAPANSLPSKV 299
Allen's_swamp_monkey             GTSSS---SCPGLASAGAAEDEQGAESDQAEPIICSSGAEAPANSLPSKV 297
Duski_titi_monkey                G--TSS--SSPGLASAGAEEG------NQPEPSICSSGAEAPANSVPSKV 289
Common_woolly_monkey             GTFASS--SSPGLASAGTEEG------DQPEPIICSSGAEAPANPLPSKV 291
Black-handed_spider_monkey       GTFSSS--SSPGLASAGAEEG------DQPEPIICSSGAEAPANSLPSKV 291
Owl_monkey                       GTFSSF--SSPGLASAGVEEG------DQPEPIICSSGAEAPANSLPSKV 291
Red-chested_mustached_monkey     GTFSSS--SSPGLASAGVEEG------DQPEPVICSSGAEAPANSLPSKV 291
Common_marmoset                  GTFSSS--S-PGLASAGVEEG------DQPEPVICSSGAEAPANSLPSKV 290
                                 *   *   * *******. *.      ::.**  ** *******. ****
```

```
Human                            PTTLMPVNTVALKVPANPASVSTVPSKLPTSSKPPGAVPSNALTNPAPSK 348
Chimpanzee                       PTTLMPVNTVALKVPANPASVSTVPSKLPTSSKPPGAVPSNVLTNPAPSK 348
Western_lowland_gorilla          PTTLMPVNTVAPKVPANPASVSTVPSKLPTSSKPPGAVPSNVLTNPAPSK 348
Bornean_orangutan                PATLMPVNTVVPKVPADPASVSTAPSKLPASSKRPGAVPSNVLTNPAPSK 348
White-cheeked_gibbon             PTTLMPVNTVALKVPANPASVSTVPSKLPTSSKPPGAVPSNVLTNPAPSK 348
Agile_gibbon                     PTTLMPVNTVALKVPANPASVSTVPSKLPTSSNPPGAVPSNVLTNPAPSK 348
Island_siamang_gibbon            PTTLMPVNTVALKVPANPASVSTVPSKLPTSSKPPGAVPSNVLTNPAPSK 348
Colobus_monkey                   PTTLMPVNTVAPKVPANPASASTLPSKLPTSSKPPGAVPS-VFTNPAPSK 343
Francois'_leaf_monkey            PTTLMPVNTVAPKVPANPASASTVPSKLPTSSKPPGTVPS-VFTNPAPSK 347
Olive_baboon                     PTTLMPVNTVAPKVPANPASASTVPSKLPTSSKPPGAVPS-VFTNPAPSK 349
Rhesus_macaque                   PTTLMPVNTVAPKVPANPASASTVPSKLPTSSKPPGTVPS-VFTNPAPSK 349
Talapoin_monkey                  PTTLMPVNTVAPKVPANPASASTVPSKLPTSSKPPGAVPS-VFTNPAPSK 348
African_green_monkey             PTTLMPVNTVAPKVPANPASASTVPSKLPTSSKPPGAVPS-VFTNPAPSK 348
Patas_monkey                     PTTLMPVNTVAPKVPANPASASTVPSKLPTSSKPPGAVPS-VFTNPAPSK 348
Allen's_swamp_monkey             PTTLMPVNTVAPKVPANPASASTVPSKLPTSSKPPGAVPS-VFTNPTPSK 346
Duski_titi_monkey                PTTLMPMNTVPPEVPANPASASTVLSKLPPSSKPPDAVPSNVLTNPAPSK 339
Common_woolly_monkey             PTTLMPVNTVPLEVPANPASASTVPSKLPPSSKPPGAVPSNVLTNPAPSK 341
Black-handed_spider_monkey       PTALMPVNTVPPEVPANPASASTVPSKLPPSSKPPGAVPSNVLTNPAPSK 341
Owl_monkey                       PTTLMPVNTVPLEVPANTASASTVPSKLPPSSKPPGAVPSNVLTNLAPSK 341
Red-chested_mustached_monkey     PTTLMPVNTVPPEVPANPASASTVTSKLPPSSKPPGAVPSNVLTNPAPSK 341
Common_marmoset                  PTTLMPVNTVPPEVPANPASASTVTSKLPPSSKPPGAVPSNVLTNPAPSK 340
                                 *::***:***  :***:.**.**  ****.**: *.:*** .:** :***

Human                            LPINSTRAGMVPSKVPTSMVLTKVSASTVPTDGSSRNEETPAAPTPAGAT 398
Chimpanzee                       LPINSTRAGMVPSKVPTSMVLTKVSASTVPTDGSSRNEETPAAPTPVGAT 398
Western_lowland_gorilla          LPINSTRAGMVPSKVPTSMVLTKVSASTVPTDRSSRNEETPAAPTPAGAT 398
Bornean_orangutan                LPINSTRAGMVPSKVPTSMVLTKVSASTVPTDRSSRNEETPAAPTPAGAT 398
White-cheeked_gibbon             LPVNSTRAGMVPSKVPTSMVLTKVSASAVPTDRSSRNEETPAAPTPAGAT 398
Agile_gibbon                     LPINSTCAGMVPSKVPTSMVLTKVSASAVPTDRSSRNEETPAAPTPAGAT 398
Island_siamang_gibbon            LPINSTHAGMVPSKVPTSMVLTKVSASAVPTDRSSRNEETPAAPTPAGAT 398
Colobus_monkey                   LPINSTRAGMVPSKVPTSMVRTKVSASTVPTDRSSRTEETPAAPTPAGAT 393
Francois'_leaf_monkey            LPINSTRAGMVPSKVPTSMVRTKVSASTVPIDRSSRTEETPAAPTPAGAT 397
Olive_baboon                     LPINSTRAGMVPSKVPTSMVRTKVSASTVPTDRSSRTEETSAAPTPAGAA 399
Rhesus_macaque                   LPINSTRAGMVPSKVPASMVRTKVSASTVPTDRSSRTEETSAAPTPAGAT 399
Talapoin_monkey                  LPINSTRAGMVPSKVPTSMVRTKVSASIVPTDRSSRTEETPAAPTPAGAT 398
African_green_monkey             LPINSTRAGMVPSKVPTSMVRTKVSASTVPTDRSSRTEETPAAPTPAGAT 398
Patas_monkey                     LPINSTRAGMVPSKVPTSMVRTKVSASTVPTDRSSRTEETPAAPTPAGAT 398
Allen's_swamp_monkey             LPINSTRAGMVPSKVPTSMVRTKVSASTVPTDRSSRTEETPAASTPAGAT 396
Duski_titi_monkey                LPINSTHAGMVPSKVPTSMVLTTGNASTVPASRSSRAEETPAAPTPAGAT 389
Common_woolly_monkey             LPINSTRAGMVPPKVPSSMVLTTGNASTVPTSRSSRAEETPAAPTPAGAT 391
Black-handed_spider_monkey       LPINSTRAGMVPSKVPSSMVLTTGNANTVPTSRSSRAEETPAAPTPAGAT 391
Owl_monkey                       LPINSTRVGIVPSKVPTSMVLTTGNASTVPTSRSSRAEENPAAPTPAGAT 391
Red-chested_mustached_monkey     LPINSTRAGMVPSKVPTSMVLTTGNASTVPTSRSSRAKETPAAPTPAGAT 391
Common_marmoset                  LPISSTRAVMVPSKVPSSMVLTTGNASTVPTSRSSRAEETPAAPTPAGAT 390
                                 **:.** . :**.***:*** *. .*. ** . *** :*..**.**.**:
```

```
Human                           -GGSSAWLDSSSENRGLGSELSKPGVLASQVDSPFSGCFEDLAISASTSL 447
Chimpanzee                      -GGSSAWLDSSSENRGLGSELSKPGVLASQVDSPFSGCFEDLAISASTSL 447
Western_lowland_gorilla         -GGSSAWLDSSSENRGLGSELSKPGVLASQVDSPFSGCFEDLAISASTSL 447
Bornean_orangutan               -GGSSAWLDSSSENGGLGSELSKPGVLASQVDSPFSGCFEDLAISASTSL 447
White-cheeked_gibbon            -GGSSAWLDSSSENGGLGSELSKPGMLASQVDSPFSGCFEDLAISASPSL 447
Agile_gibbon                    -GGSSAWLDSSSENGGLGSELSKPGVLASQVDSPFSGCFEDLAISASTSL 447
Island_siamang_gibbon           -GGSSAWLDSSSENGGLGSELSKPGVLASQVDSPFSGCFEDLAISASTSL 447
Colobus_monkey                  -GGRSAWLDSSSENGSFELELSKPGMLVSQADSQFSGCSEDLAISASTSL 442
Francois'_leaf_monkey           -GGCSAWLDSSSENGSFELELSKPGMLVSQADSQFSGCSEDLAISASTSL 446
Olive_baboon                    -GGRSAWLDSSSENGGFESELSKPGILVSQADSQFSGCSEDLAISASTSL 448
Rhesus_macaque                  -GGRSAWLDSSSENGGFESELSKPGILVSQADSQFSGCSEDLAISASTSL 448
Talapoin_monkey                 -GGRSAWLDSSSENGGFESELSKPGMLVSQPDSQFSGCSEDLAISASTSL 447
African_green_monkey            -GGRSAWLDSSSENGGFESELSKPGMLVSQADSQFSGCSEDLAISASTSL 447
Patas_monkey                    -GGRSAWLDSSSENGGFESELSKPGMLVSQADSPFSGCFEDLAISASTSL 447
Allen's_swamp_monkey            TGGRSAWLDSSSENGGFESELSKPGMLVFQADSQFSGCSEDLAISASTSL 446
Duski_titi_monkey               -GGSSAWLDKNSENGGLGSELSKPGILVSQVDSLFSGCSEDLAISASSSL 438
Common_woolly_monkey            -GGSSAWLDNSSENGGLGSELSKPGMLVSQVDSLFSGCSEDLAISASSSL 440
Black-handed_spider_monkey      -GGSSAWLDNSSENGGLGSELSKPGMLVSQVDSLFSGCSEDLAISASSSL 440
Owl_monkey                      -GGSSAWLDNSSENGGLGSELSKPGILVSQVDSLFSGCSEDLAISASTSL 440
Red-chested_mustached_monkey    -GGSSAWLDNSSENGGLGSELSKPGMLVSQVDSLFSGCSEDLAISASSSL 440
Common_marmoset                 -GGSSAWLDNSSENGGLGSELSKPGMLVSQVDSLFSGCSEDLAISASSSL 439
                                 ** *****..*** .:  ******:*. * ** **** ********.**

Human                           GMGPCHGPEENEYKSEGTFGIHVAENPSIQLLEGNPGPPADPDGGPRPQA 497
Chimpanzee                      GMGPCHGPEENEYKSEGTFGIHVAENPSIQLLEGNPGPPADPDGGPRPQT 497
Western_lowland_gorilla         GMGPCHGPEENEYKSEGTFGIHVAENPSIQLLEGNPGPPADPDGGPRPQT 497
Bornean_orangutan               GIGPCHGPEENEYKSEGTFGIHVAENPSIQLLEGNPGPPTDPECGPRPQT 497
White-cheeked_gibbon            GMGPCHGPEENEYKSEGTFGIHVAENPSIQLLEGNPGPPVDPEGGPRPQT 497
Agile_gibbon                    GMGPCHGPEENEYKSEGTFGIHVAENPSIQLLEGNPGPPVDPEGGPRPQT 497
Island_siamang_gibbon           GMGPCHGPEENEYKSEGTFGIHVAENPSIQLLEGNPGPPVDPEGGPRPQT 497
Colobus_monkey                  GMGPCHGPEENEYKSEGTFGIHVAENPSIQLMEGNPGPPADPQSSPRSHI 492
Francois'_leaf_monkey           GMGPCHGPEENEYKSEGTFGIHVAENPSIQLK-GIPGPPADPQGGPRPHI 495
Olive_baboon                    GMGPCHGPEENEYKSEGTFGIHVAENPSIQLMEGNPGPPADPQGGPRPHI 498
Rhesus_macaque                  GMGPCRGPEENEYKSEGTFGIHVAENPSIQLMEGNPGPPADPQGGPRPHI 498
Talapoin_monkey                 GMGPCHGPEENEYKSEGTFGIHVAENPSIQLMEGNPGPPADLQGGPRPHT 497
African_green_monkey            GMGSCHGPEENEYKSEGTFGIHVAENPSIQLMEGNPGPPAGPQGGPRPHV 497
Patas_monkey                    GMGPCHGPEENEYKSEGTFGIHVAENPSIQLMEGNPGPPADPQGGPRPHV 497
Allen's_swamp_monkey            GMGPCHGPEENEYKSEGTFGIHVAENPSIQLMEGNPGPPADPQGGPRPHI 496
Duski_titi_monkey               DVGPCHGPEENEYKSEGTFEIHVAENPSIQLLEGNPGPPADLEGGPRPHT 488
Common_woolly_monkey            GVGPCHGPEENEYKSEGTFGIHVAEDPSIQLLEGNPGLPADLEGGPRPHT 490
Black-handed_spider_monkey      GVGPCHGPEENEYKSEGTFGIHVAEDPSIQLLEGNPGLPADLEGGPRPHT 490
Owl_monkey                      GMGPCHGPEENEYKSEGTFGIHVAENPSIQLLEANPGPPADLEGGPRPHT 490
Red-chested_mustached_monkey    GMGPCHGPEENEYKSEGTFGIHVAENPSIQLLEGNPGPPADLEGGPRPHA 490
Common_marmoset                 GVGPCHGPEENEYKSEGTFGIHVAENPSIQLLEGNPGPPADLEGGPRPHT 489
                                .:*.*:************* *****:*****  . ** *.. : .**.:
```

```
Human                             DRKFQEREVPCHRPSPGALWLQVAVTGVLVVTLLVVLYRRRLH 540
Chimpanzee                        DRKFQEREVPCHRPSPGALWLQVAVTGVLVVTLLVVLYRRRLH 540
Western_lowland_gorilla           DRKFQEREVPCHRPLPGALWLQVAVTGVLVVTLLVVLYRRRLH 540
Bornean_orangutan                 NQKFQEGEVPCHRPSPGALWLQVAVTGVLVVTLLVVLYRRRLH 540
White-cheeked_gibbon              GRKFQEGEVPCHRPSPGALWLQVAVAGVLAVTLLVVLYRRRLH 540
Agile_gibbon                      GRKFQEGEVPCHRPSPGALWLQVAVAGVLAVTLLVVLYRRRLH 540
Island_siamang_gibbon             GRKFQEGEVPCHRPSPGALWLQVAVAGVLAVTLLVVLYRRRLH 540
Colobus_monkey                    DQKFQEREVPCHRPSPGALWLQAAVAGVLVVTLLVAMYRRRLH 535
Francois'_leaf_monkey             DQKFQEWEVPCHRPSPGALWLQAAVAGVLVVTLLVAMYRRRLH 538
Olive_baboon                      DQKFQEREVPRHRPSPGALWLQAAVAGVLVVTLLVAMYRRRLH 541
Rhesus_macaque                    DQKFQEREGPCHRPSPGALWLQAAVAGVLVVTLLVAMYRRRLH 541
Talapoin_monkey                   DQKFQEREVPCHRPSPGALWLQAAVAGVLVVTLLVAMYRRRLH 540
African_green_monkey              DQKFQKREVPCHRPSPGALWLQAAVAGVLVVTLLVAMYRRRLH 540
Patas_monkey                      DQKFQKREVPCHRPSPGALWLQAAVAGVLVVTLLVAMYRRRLH 540
Allen's_swamp_monkey              DQKFQEREMPCHRPSPGALWLQAAVAGVLVVTLLVAMYRRRLH 539
Duski_titi_monkey                 NQKFQDEEAPCHWSSLGSPWLQAAVAGVLAAILLAMLYQRRQL 531
Common_woolly_monkey              DQKFQDEEVPCHWSSLGSPWLQAAMAGVLAAILLAVLYQRRQL 533
Black-handed_spider_monkey        DQKFQDEEAPCHWSSLGSPWLQAAVAGVLAAILLAVLYQRRQL 533
Owl_monkey                        DQKFQDEEVPCHWSSLSSPWLQAAVAGMLAAILLAVLYQRRQL 533
Red-chested_mustached_monkey      DQKFQDEEVPCHWSSLGSPWLQAAVAGVLAAILLAVLYQRRQL 533
Common_marmoset                   DQKFQDEEVPCHWSSLGSPWLQAAVAGVLAAILLAVLYQRRQL 532
                                   .:***. * * * .  .: ***.*::*:*.. **. :*:**
```
